# A phylogenetic survey of myotubularin genes of eukaryotes: distribution, protein structure, evolution, and gene expression

**DOI:** 10.1186/1471-2148-10-196

**Published:** 2010-06-24

**Authors:** David Kerk, Greg BG Moorhead

**Affiliations:** 1 Department of Biological Sciences, University of Calgary, 2500 University Drive N.W., Calgary, Alberta, T2N 1N4, Canada

## Abstract

**Background:**

Phosphorylated phosphatidylinositol (PtdIns) lipids, produced and modified by PtdIns kinases and phosphatases, are critical to the regulation of diverse cellular functions. The myotubularin PtdIns-phosphate phosphatases have been well characterized in yeast and especially animals, where multiple isoforms, both catalytically active and inactive, occur. Myotubularin mutations bring about disruption of cellular membrane trafficking, and in humans, disease. Previous studies have suggested that myotubularins are widely distributed amongst eukaryotes, but key evolutionary questions concerning the origin of different myotubularin isoforms remain unanswered, and little is known about the function of these proteins in most organisms.

**Results:**

We have identified 80 myotubularin homologues amidst the completely sequenced genomes of 30 organisms spanning four eukaryotic supergroups. We have mapped domain architecture, and inferred evolutionary histories. We have documented an expansion in the Amoebozoa of a family of inactive myotubularins with a novel domain architecture, which we dub "IMLRK" (inactive myotubularin/LRR/ROCO/kinase). There is an especially large myotubularin gene family in the pathogen *Entamoeba histolytica*, the majority of them IMLRK proteins. We have analyzed published patterns of gene expression in this organism which indicate that myotubularins may be important to critical life cycle stage transitions and host infection.

**Conclusions:**

This study presents an overall framework of eukaryotic myotubularin gene evolution. Inactive myotubularin homologues with distinct domain architectures appear to have arisen on three separate occasions in different eukaryotic lineages. The large and distinctive set of myotubularin genes found in an important pathogen species suggest that in this organism myotubularins might present important new targets for basic research and perhaps novel therapeutic strategies.

## Background

Phosphatidylinositol (PtdIns) phospholipids are quantitatively minor but functionally significant membrane lipid components which have been shown to be involved in regulating diverse aspects of cellular function, such as proliferation, survival, growth, cytoskeletal reorganization, and various membrane trafficking events. The inositol ring can be phosphorylated at the D3, D4 or D5 position to produce a set of seven distinct phosphorylated derivatives, which are preferentially located in various cellular membranes or microdomains, specifying their identity, and mediating cellular functions by recruiting various effector proteins with specialized lipid-binding domains [1]. The homeostasis of these phosphorylated PtdIns lipids is mediated by a number of specific kinases and phosphatases.

Myotubularins are members of the protein tyrosine phosphatase (PTP) superfamily, which feature a characteristic HCX(5)R catalytic motif, where the cysteine is the catalytic residue, the histidine is important for the nucleophilic properties of the cysteine, and the arginine is important in coordinating the substrate phosphate group. Myotubularins have been shown to be specific lipid phosphatases, cleaving the D3 phosphate from PtdIns3P and PtdIns(3,5)P2. There is a large myotubularin family in humans (14 genes) which encode both catalytically active and inactive members. Mutations in either active or inactive members of this family bring about human disease, which involves chiefly skeletal muscle (X-linked myotubular myopathy [XLMTM]) or peripheral neurons (Charcot-Marie-Tooth [CMT] neuropathies) [2-4]. Previous phylogenetic studies have reported the presence of myotubularin genes in plants, fungi and some protists, with the latter group only containing both active and inactive forms [2,5].

This study presents a systematic survey of myotubularin genes in a large number of completely sequenced eukaryotic genomes, representing a broad array of taxonomic groups. Most genomes contain one to a few myotubularin genes, though they are absent in certain groups. The evidence is consistent with the independent appearance of inactive myotubularin genes, featuring novel domain combinations, in different taxonomic groups. The greatest expansion of the myotubularin gene family yet observed occurs in the pathogenic species *Entamoeba histolytica*. Functional evidence derived from published gene expression studies indicates that these genes may be important in pathogen transmission and host infection.

## Results

### Phylogenetic Distribution, Gene Evolution, Domain Architecture

**Figure 1:**
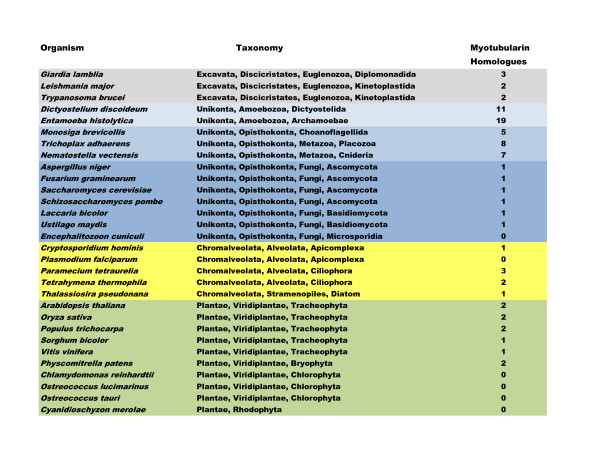
**Myotubularin Homologues in Eukaryotic Protein Datasets**. Listed is the set of eukaryotic species with completely sequenced genomes whose protein datasets were searched for myotubularin homologues. The number of myotubularin homologues detected for each genome is listed. Color coding: Excavates (gray); Unikonts, Amoebozoa (pale blue); Unikonts, Choanoflagellates/Metazoa (medium blue); Unikonts, Fungi (dark blue); Chromalveolates (yellow); Plantae (green). Taxonomy was taken from: NCBI Taxonomy Browser http://www.ncbi.nlm.nih.gov/Taxonomy/Browser/wwwtax.cgi?mode=Root; Tree of Life http://tolweb.org/Eukaryotes/3; and Koonin, 2010 [8]. The URLs for downloading of all organismal datasets, and the original publication references, are given in Additional File 4. Figure design after Gazave et al., 2009 [68].

Recent work in eukaryotic systematics has increasingly defined large organismal "supergroups" encompassing many traditional smaller groups [6-8]. We have conducted a broad survey of fully sequenced genomes amongst these large organismal groups for the presence of myotubularin gene homologues. Only the Rhizaria were excluded as there is as yet no completed genome in that group. Our results are summarized in Figure 1. We searched 30 genomes, and identified 80 sequences. We found that myotubularin genes are nearly ubiquitous in eukaryotes, being readily identifiable in all the major eukaryotic groups and in all genomes examined with the notable exception of the obligate intracellular parasites *Encephalitozoon cuniculi *(Microsporidia) and *Plasmodium falciparum *(Apicomplexa) and eukaryotic algae, both red (*Cyanidioschyzon merolae*) and green (*Ostreococcus *sp., *Chlamydomonas reinhardtii*). Most organisms (19 out of 24 species with myotubularins) posses one to three genes. The notable exception to this general pattern occurs in members of the Unikonta (Amoebozoa, Choanoflagellida, Metazoa) (for more information on organisms, see the Tree of Life project [9]).

**Figure 2:**
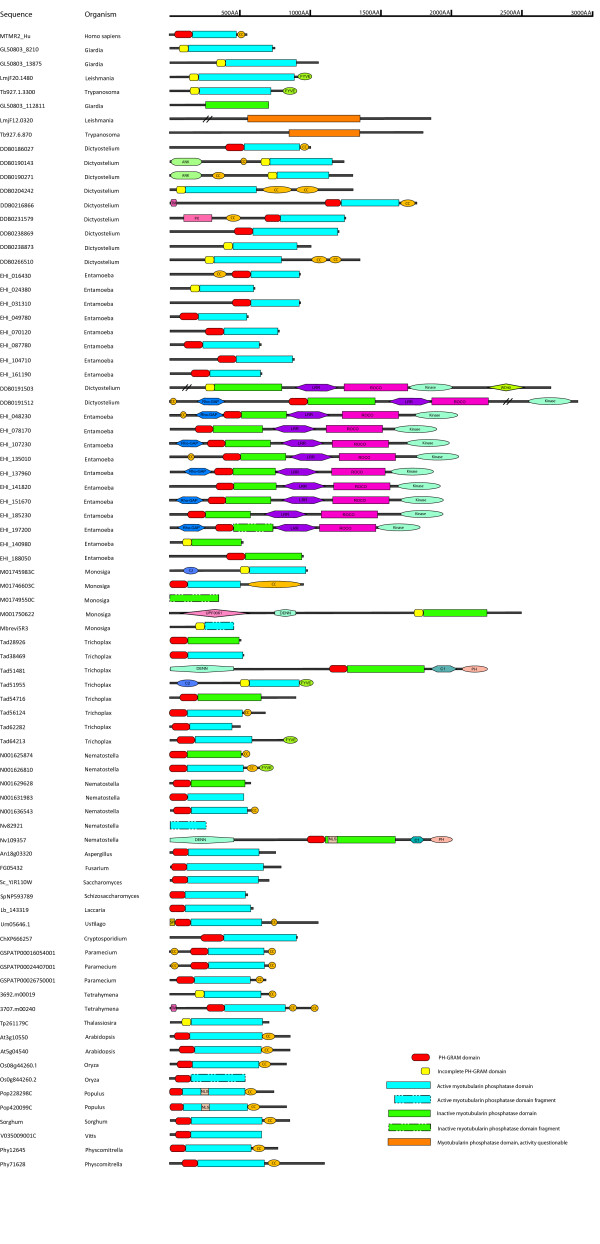
**Myotubularin Protein Domain Architecture**. Candidate myotubularin homologue sequences were obtained by searching the protein datasets of fully sequenced eukaryotic genomes, as detailed in Methods. PH-GRAM, myotubularin phosphatase, and other structural domains were identified as detailed in Methods. To conserve space, organisms are identified in the Figure as genus names only. Organisms are grouped in this figure by taxonomic category as in Figure 1. The domain architecture of human MTMR2 is given for orientation purposes. The organisms are as follows: *Homo sapiens*, *Giardia lamblia*, *Leishmania major*, *Trypanosoma brucei*, *Dictyostelium discoideum*, *Entamoeba histolytica*, *Monosiga brevicollis*, *Trichoplax adhaerens*, *Nematostella vectensis*, *Aspergillus niger*, *Fusarium graminearum*, *Saccharomyces cerevisiae*, *Schizosaccharomyces pombe*, *Laccaria bicolor*, *Ustilago maydis*, *Cryptosporidium hominis, Paramecium tetraurelia*, *Tetrahymena thermophila*, *Thalassiosira pseudonana*, *Arabidopsis thaliana*, *Oryza sativa*, *Populus trichocarpa, Sorghum bicolor, Vitis vinifera*, *Physcomitrella patens*. The complete set of sequences illustrated in the Figure, along with database accession numbers, is available as Additional File 5. As detailed in Methods, some incomplete candidate myotubularin sequences with annotation mistakes were corrected with additional sequence, and are denoted with the suffix "C" in the Figure. Sequence "Mbrevi5R3" was assembled manually from individual genomic sequence reads unincorporated into scaffolds, through use of TBLASTN against *Monosiga *genomic DNA with *Nematostella *myotubularin homologue query sequences.

We utilized domain-searching strategies detailed in Methods to determine the molecular architecture of myotubularin gene encoded proteins. The results are presented in Figure 2. It is apparent that nearly all myotubularin proteins contain both a myotubularin phosphatase domain and a PH-GRAM domain (**P**leckstrin-**H**omology, **G**lucosyltransferases, **R**ab-like GTPase **A**ctivators and **M**yotubularins). In studies of animal myotubularin proteins it has been shown that the PH-GRAM domain binds phosphoinositide lipids, and confers both specific subcellular localization and regulation of the phosphatase domain [10]. The nearly constant presence of the PH-GRAM domain in myotubularins across a broad range of organisms suggests that this domain architecture was established early in eukaryotic evolution. We observed, however, that there were a number of sequences where complete PH-GRAM domains with the characteristic architecture observed in human proteins could not be detected, despite the use of the most sensitive structural analysis methods available (see Figure 2). This indicates that PH-GRAM domain sequences can be very divergent, which we also noted in multiple sequence alignments including the PH-GRAM domain region (see the full myotubularin sequences alignment presented as Additional File 1). This suggests that although the architectural coupling of a PH-GRAM along with a myotubularin phosphatase domain is a standard feature of these proteins, the specific molecular properties and functions of the PH-GRAM domains have the potential to be quite diverse and distinct.

**Figure 3:**
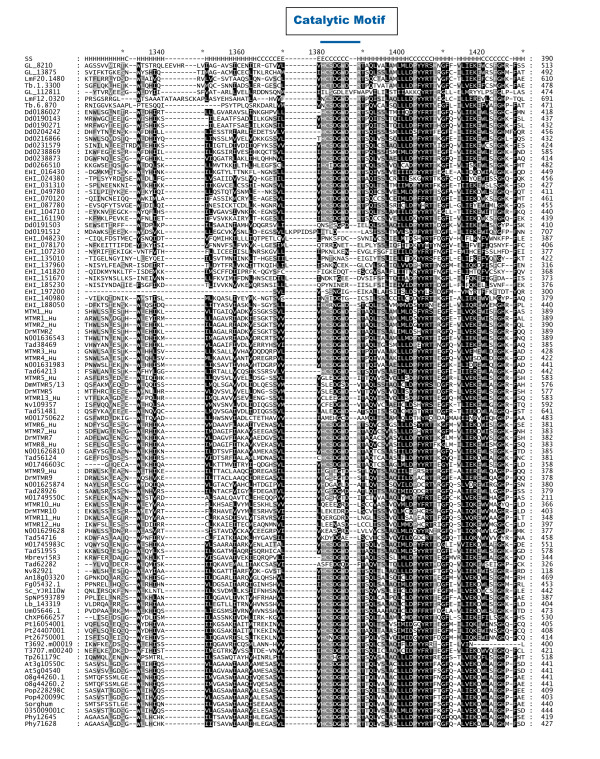
**Alignment of Catalytic Region of Myotubularin Domain Sequences**. Human myotubularin sequences were obtained from the literature and database keyword searches. For the other species represented, candidate myotubularin sequences were obtained by either utilizing BLASTP at NCBI [69] or searching the protein datasets of fully sequenced eukaryotic genomes, as detailed in Methods. The contiguous sequence encompassing the PH-GRAM domain and the myotubularin phosphatase domain was identified as detailed in Methods, and multiple alignment was performed as detailed in Methods. Sequences are presented in the general order of taxonomic groups specified in Figure 1, with the exception that Choanoflagellate/Metazoan sequences are grouped according to phylogenetic classification as in Table 2. Non-human sequences in the alignment are generally designated by an organism prefix followed by numerals. This Figure presents the portion of the alignment around the catalytic loop region. The full alignment is presented as Additional File 1. At the top of the alignment, in the line designated "SS", is depicted the secondary structure of the solved structure of human MTMR2 (PDB:1LW3[70]). "H" indicates alpha helix, "C" indicates random coil, "E" indicates beta strand. Above the alignment a blue bar (positions 1380 - 1389) indicates the location of the catalytic motif of the myotubularin phosphatase domain (HCSDGWDR for active subunits). The sequences for all myotubularin homologues, along with their database accession numbers, and designations used in this Figure, are presented in Additional File 5. The URLs for all web sites used to obtain organism specific databases, plus original literature citations, are presented in Additional File 4.

The catalytic loop signature of human myotubularins is: HCSDGWDR [2]. Inspection of the myotubularin sequence alignment presented in Figure 3 shows that this is found invariant in most of the myotubularin sequences, indicating that they all share a common local active site architecture and catalytic mechanism. One of the notable features of human myotubularins is the presence of several catalytically inactive subunits, resulting from mutations to the key catalytic cysteine and arginine residues in the catalytic loop region. It has been previously noted that myotubularin genes with apparently inactive catalytic loop signatures can be observed in *Giardia *and *Dictyostelium*, suggesting that inactive subunits arose early in evolution [2,5]. Our work confirms these findings, and sheds further light on the origin of these sequences. Three Excavate myotubularin sequences lack a PH-GRAM domain, the only sequences we observed with this characteristic (see Figure 2). *Giardia *sequence GL50803_112811 lacks both the cysteine and arginine residues from the catalytic loop region (see Figure 3). *Leishmania *sequence LmjF12.0320 and *Trypanosoma *sequence Tb927.6.870 each possess both the cysteine and the arginine, but lack the histidine preceding the cysteine. Since this histidine is universally conserved in active PTP phosphatases, and has been shown to be important in the catalytic mechanism by altering the nucleophilic properties of the neighboring cysteine [11], it is likely that these proteins are also catalytically inactive. The lack of a PH-GRAM domain, unique to these Excavate inactive myotubularins, suggests that they comprise a single gene lineage.

#### Amoebozoan IMLRK (Inactive Myotubularin/LRR/ROCO/Kinase) Genes and Proteins

The amoebozoans *Dictyostelium *and *Entamoeba *each have a large number of myotubularin homologues (see Figure 1 and Figure 2). *Dictyostelium *has nine active myotubularin subunits, and *Entamoeba *has eight. In addition, there are a number of inactive myotubularin subunits. The *Dictyostelium *gene pats1 (encoding sequence DDB0191503) was previously incorrectly reported to contain an active myotubularin domain [12]. In addition, this protein contains a LRR domain, a recently described ROCO domain [13,14] (comprised of a ROC [Ras of complex proteins] and COR [C-terminal of ROC] region), and a protein kinase domain. The LRR/ROCO/kinase architecture was also known to be shared by *Dictyostelium *sequence DDB0191512, which also has an N-terminal Rho-GAP domain. By use of a sensitive myotubularin-sequence based HMM search strategy, we found that this sequence also contains an inactive myotubularin domain. Further application of this HMM search revealed that *Entamoeba *contains eleven proteins with divergent, but clearly recognizable inactive myotubularin domains (EHI_140980, EHI_137960, EHI_185230, EHI_048230, EHI_151670, EHI_107230, EHI_135010, EHI_141820, EHI_078170, EHI_197200, EHI_188050). Our findings confirm and extend previous observations [5]. Further examination of the domain architecture of the newly discovered *Entamoeba *inactive myotubularin sequences revealed that 9 of them also showed significant similarity to the solved structures of LRR proteins and protein kinases, and weaker but still significant similarity to the solved structure of a bacterial ROCO protein (PDB: 3dpu_A) [15] as detected by both the FFAS03 (**F**old and **F**unction **A**ssignment **S**ystem ) sequence:profile technique, and the HHPred (**H**MM-**H**MM {**H**idden **M**arkov **M**odel} structure **p**rediction) profile:profile technique. This indicated that these nine proteins might also share the inactive myotubularin/LRR/ROCO/kinase architecture previously detected in *Dictyostelium *sequences. We suggest the acronym "IMLRK" to refer to this somewhat cumbersome domain architecture.

**Figure 4:**
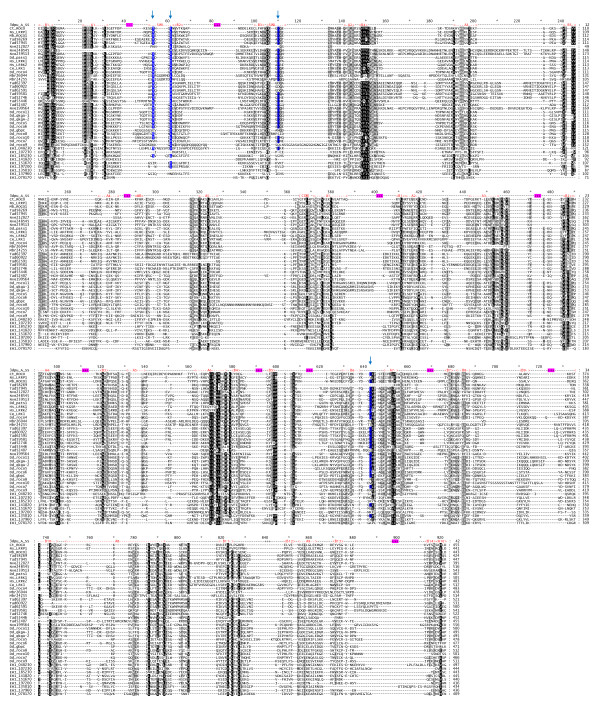
**Alignment of ROCO Domain Sequences**. ROCO domain sequences were identified and aligned as detailed in Methods. The alignment presents the sequences of six ROCO domain reference proteins: "Ct_ROCO" (*Chlorobium tepidum*); "Ns_LRRP1" (*Nostoc *sp. PCC 7120); "Mb_ROCO1" (*Methanosarcina barkeri *str. Fusaro); "Hs_LRK1" (*Homo sapiens*); "Hs_LRRK2" (*Homo sapiens*); and "Ce_LRK1" (*Caenorhabditis elegans*). Most of the other sequences are designated by an organism prefix, followed by a number from the appropriate organism-specific protein database. Several *Dictyostelium *("Dd") sequences are referred to by their gene names. Further information about the reference and candidate ROCO sequences, including organism prefixes and database accession numbers, are provided in Additional File 6. Conserved residues shown by Gotthardt et al. [15] as being critical to the functioning of both the bacterial protein and the human ROCO protein homologue LRRK2 are marked with blue lettering outlining and blue arrows. These positions are as follows: "T484" (our T53); "L487" (our L61); "G518" (our G110); and "Y804" (our Y642). Above the alignment is shown in red the secondary structure of the solved structure of the ROCO domain of *Chlorobium tepidum *(PDB: 3dpu_A). "A" indicates alpha helix, "B" indicates beta strand, and arrowhead symbols ("<" and ">") denote the beginning and ending of secondary structure regions. The functionally important "Switch I" ("SW1") and "Switch II" ("SW2") regions are indicated. Areas with "+++" symbols in purple represent poorly aligned sequence regions which have been edited from the alignment. The initial sequence region (positions 1-365) represents the ROC domain. The beginning of the COR domain is indicated.

To confirm the identity of the ROCO domains of these *Entamoeba *sequences we performed iterative multiple sequence alignments, HMM construction, database searches and realignment, to assemble the data presented as Figure 4. During this process, we identified several previously unreported ROCO proteins (2 from *Monosiga *and 9 from *Trichoplax*). The alignment presents a comparison between our set of newly identified ROCO domain sequences and those from previously characterized *Dictyostelium *proteins. In their report of the solved structure of a bacterial ROCO protein, Gotthardt et al. [15] identified residues important to both the function of the bacterial protein, and animal ROCO protein homologues. These include residues in the ROC domain important for GTPase binding and residues in both the ROC and COR domains important for domain interactions and GTPase activity (see Legend to Figure 4). It is evident by inspection of the alignment in Figure 4 that on the whole, conservation of this critical residue set for the *Entamoeba *IMLRK sequences is poor. Despite the overall apparent similarity of these sequences to the rest of the comparison set, several of the *Entamoeba *sequences have deletions in these critical residues, and would therefore presumably lack GTPase activity. Only one *Entamoeba *sequence (EHI_048230) has a set of residues which might confer enzymatic activity.

**Figure 5:**
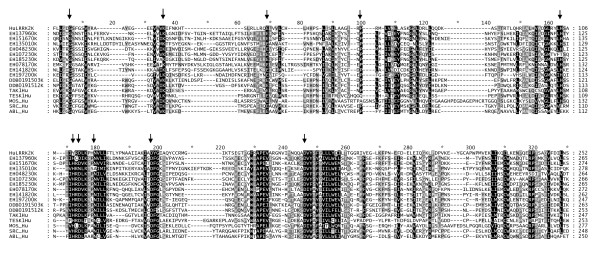
**Alignment of ROCO Kinase Domain Sequences**. Myotubularin homologue sequences were identified as detailed in Methods. The protein kinase domains of these sequences were identified by searching at the NCBI CDD [40,41]. "EHI" refers to *Entamoeba histolytica *myotubularin proteins, "DDB" refers to *Dictyostelium discoideum *myotubularin proteins, "Hu" refers to *Homo sapiens *proteins. Reference protein kinase sequences were obtained from the literature and keyword search as follows: "HuLRRK2" (GenBank: NP_940980); "TAK1Hu" (PDB: 2EVA_A); "TESK1Hu" (GenBank: NP_006276.2); "MOS_Hu" (GenBank: NP_005363.1); "SRC_Hu" (Swiss-Prot:P12931); "ABL_Hu" (Swiss-Prot:P00519). The figure has arrows marking the positions of functionally important residues, as defined by Kannan et al. [18].

Comparison with sequence models at NCBI CDD (**C**onserved **D**omain **D**atabase) indicates that the protein kinase domains of the *Entamoeba *IMLRK proteins resemble both Ser-Thr and Tyr kinases (see Table 1). This is consistent with previous characterization of kinase domains in ROCO proteins as being of the TKL (**T**yrosine **k**inase-**l**ike) group [16,17]. We performed a multiple sequence alignment with these kinase domains, which is presented in Figure 5. We examined the group of ten important functional sequence positions characterized by Kannan et al. [18]. For 7 of the 9 Entamoeba sequences, all of these critical functional residues are conserved. The exceptions are: EHI_107230, where there is an H to T mutation at the position corresponding to PKA (**P**rotein **K**inase **A**) H158; and EHI_135010, where there is a D to N mutation at the position corresponding to PKA D166, and an N to A mutation at the position corresponding to PKA N171. Thus we would predict that nearly all of these sequences are catalytically active [18].

**Table 1:** ROCO Kinase Domain CDD Hits

Query Sequence	NCBI CDD Hits	E values
EHI_137960	cd00180, S_TKc cd00192, PTKc	4.00E-35 2.00E-32

EHI_151670	cd00192, PTKc cd00180, S_TKc	8.00E-38 1.00E-29

EHI_135010	cd00192, PTKc cd00180, S_TKc	5.00E-33 7.00E-28

EHI_048230	cd00180, S_TKc cd00192, PTKc	1.00E-28 5.00E-26

EHI_107230	cd00192, PTKc cd00180, S_TKc	1.00E-35 2.00E-26

EHI_185230	cd00180, S_TKc cd00192, PTKc	8.00E-34 3.00E-32

EHI_078170	cd00192, PTKc cd00180, S_TKc	7.00E-37 1.00E-31

EHI_141820	cd00192, PTKc cd00180, S_TKc	1.00E-45 3.00E-41

EHI_197200	cd00192, PTKc cd00180, S_TKc	2.00E-35 3.00E-31

DDB0191503	cd00192, PTKc cd00180, S_TKc	2.00E-52 1.00E-38

DDB0191512	cd00192, PTKc cd00180, S_TKc	3.00E-40 3.00E-38

HuLRRK2	cd00180, S_TKc cd00192, PTKc	3.00E-38 5.00E-36

HuPKAalpha1	cd00180, S_TKc cd00192, PTKc	3.00E-82 3.00E-22

SRC_Hu	cd00192, PTKc cd00180, S_TKc	3.00E-104 9.00E-51

Myotubularin homologue sequences were identified, and protein kinase domains identified, as described in Methods. Protein kinase domains were subjected to analysis at NCBI CDD [40,41]. This table summarizes the returns for those searches - the query sequences, the hit models, and E values. Models: cd00180, S_TKc (Serine/Threonine protein kinases, catalytic domain); cd00192, PTKc (Catalytic Domain of Protein Tyrosine Kinases).

Five of the newly discovered *Entamoeba *sequences have predicted N-terminal Rho-GAP (Rho-**G**TPase **A**ctivating **P**rotein) domains. Of these five domains, however, only one (that for EHI_048230) is strong enough to appear in a domain search at NCBI CDD with default settings, indicating that it is probably enzymatically active. The enzymatic activity of the other four domains is questionable, due to their evident sequence divergence. These five sequences with an N-term Rho-GAP domain resemble the architecture of the *Dictyostelium *gene roco9 (protein sequence DDB0191512), and it is possible that they represent a distinct gene lineage.

The myotubularin domains of the IMLRK proteins are divergent, as is evident by inspection of our sequence alignments (Figure 3 and Additional File 1). The *Entamoeba *IMLRK proteins have all suffered deletion of the α14 region of the phosphatase domain (positions 1701 - 1706 of our reference alignment [Additional File 1]). Sequence EHI_197200 is clearly the most divergent of the group. It is also missing the α8 and α9 regions, and the C-terminus of the phosphatase domain (from α14 on). In summary, the IMLRK domain architecture is distinctive, being seen in no other taxonomic group besides the Amoebozoa, which suggests that the origin of these genes comprises a second, independent event in myotubularin gene evolution.

Finally, we attempted to determine, by multiple sequence alignment and phylogenetic tree analysis (data not shown) the possible origin of the two inactive *Entamoeba *myotubularins without a ROCO domain. EHI_188050 appears to be closely related to an active subunit (EHI_104710), and has therefore probably recently suffered inactivating mutations. The origin of EHI_140980 is more obscure - it does not appear to be closely related to any of the other inactive *Entamoeba *myotubularins.

#### Myotubularins in the Choanoflagellate/Metazoan Assemblage

Previous phylogenetic analyses [2,3] of myotubularin sequences from human, other vertebrates, and a collection of invertebrates defined six similarity clusters - three composed of catalytically active subunits (in human: ["M1 Group": MTM1 {Myotubular myopathy}, MTMR1 {MTM-related}, MTMR2]; ["R3 Group": MTMR3, MTMR4]; ["R6 Group": MTMR6, MTMR7, MTMR8]) and three composed of catalytically inactive subunits (in human: ["R5 Group": MTMR5, MTMR13]; ["R9 Group": MTMR9]; ["R10 Group": MTMR10, MTMR11, MTMR12]). We have extended this analysis by finding previously unreported myotubularin homologues in the metazoans *Nematostella *and *Trichoplax*, and the choanoflagellate *Monosiga*. Our results are presented in Table 2, along with Bayesian and Maximum Likelihood clade supports for each group. Bayesian support is high for all groups, with the mean posterior probability exceeding 0.90 in every case. Bootstrap support in Maximum Likelihood is weaker and more variable, depending more on details of alignment composition, but nevertheless the mean exceeds 80% for each group. Despite repeated attempts using distinct input alignments, data-transformation techniques (i.e. identifying and removing rapidly evolving sites [6]) and amino acid substitution models, we were unable to obtain consistent tree topologies with high support for deep interior branch points. This indicates a high degree of sequence divergence of the several myotubularin sub-types.

**Table 2:** Placement of Myotubularin Homologue Sequences into Phylogenetic Similarity Clusters

"M1"(+)	"R3"(+)	"R5"(-)	"R6"(+)	"R9"(-)	"R10"(-)
0.998 ± 0.0005	0.998 ± 0.0005	0.958 ± 0.032	1.000 ± 0.000	0.990 ± 0.014	0.913 ± 0.118
84.8 ± 10.5	91.5 ± 15.0	87.5 ± 8.2	98.5 ± 1.3	95.3 ± 2.5	89.0 ± 19.4
MTM1_Hu	MTMR3_Hu	MTMR5_Hu	MTMR6_Hu	MTMR9_Hu	MTMR10_Hu
MTMR1_Hu	MTMR4_Hu	DrMTMR5	MTMR7_Hu	DrMTMR9	DrMTMR10
MTMR2_Hu	N001631983	MTMR13_Hu	DrMTMR7	N001625874	MTMR11_Hu
DrMTMR2	Tad64213	Nv109357	MTMR8_Hu	Tad28926	MTMR12_Hu
N001636543		Tad51481	N001626810	M01749550C	N001629628
Tad38469		M001750622	Tad56124		Tad54716
			M01746603C		

Myotubularin homologue sequences from human ("Hu") and *Danio rerio *("Dr") were obtained from the literature and database keyword searches. For the other species represented, candidate myotubularin sequences were obtained by searching the protein datasets of fully sequenced eukaryotic genomes, as detailed in Methods. The prefix "N" or "Nv" followed by a numeral denotes sequences from *Nematostella vectensis*, "Tad" followed by a numeral denotes sequences from *Trichoplax adhaerens*, and "M" followed by a numeral denotes sequences from *Monosiga brevicollis*, (except "Mbrevi5R3" which is also from Monosiga). The sequences were aligned as detailed in Methods. Phylogenetic trees were inferred as detailed in Methods. Shown are the composition and clade support for the six similarity groups previously identified [2,3]. The plus and minus symbols (+,-) indicate the presence of enzymatically active or inactive catalytic loop sequence signatures, respectively. For each group, the number above the line indicates the Bayesian clade support (posterior probability), and the number below the line indicates the bootstrap support (out of 100 total replicates) in Maximum Likelihood (ML). Results presented are the mean (± SD) of four distinct alignments.

Sequences Tad51955 and M01745983 consistently clustered together as a distinct "new clade". The mean Bayesian posterior probability was: 0.998 (± 0.0005) (n = 4). The mean bootstrap support in Maximum Likelihood (ML) was: 97.5 (± 0.577) (n = 4).

Sequences Nv82921, Tad62282 and MBrevi5R3 could not be placed into a similarity group with confidence.

The domain architecture data presented in Figure 2 are for the most part consistent with the placement of the new myotubularin homologues into similarity clusters based on phylogenetic tree inference data. All of the new sequences placed into similarity groups have a full PH-GRAM domain, and a myotubularin phosphatase domain (predicted to be active or inactive) consistent with their class placement. The myotubularins of the "R5" group characteristically possess a DENN (**D**ifferentially **E**xpressed in **N**eoplastic versus **N**ormal cells) domain N-terminal to the PH-GRAM domain, and a PH (**P**lekstrin **H**omology) domain C-terminal to the phosphatase domain. This is true for the new sequences Tad51481 (*Trichoplax*) and Nv19357 (*Nematostella*). However sequence M001750622 (*Monosiga*) has an additional domain of unknown function at the extreme N-terminus, and lacks the C-terminal PH domain. This may indicate that the stable "R5" subunit domain architecture had not yet been achieved at this early stage of myotubularin gene evolution. Myotubularins of the "R3" group characteristically have a FYVE domain (**F**ab 1, **Y**O1B, **V**ac 1, and **E**EA1 (early endosome antigen 1)) C-terminal to the phosphatase domain. This is true for sequence Tad64213 (*Trichoplax*). However, sequence N001631983 (*Nematostella*), also classified as an R3 member, lacks this domain. Furthermore, sequence N001626810 (*Nematostella*), classified as a member of the "R6" group, has a C-terminal FYVE domain. This is characteristically absent from the members of the R6 group, and for example, is absent from sequence Tad56124 (*Trichoplax*), also classified in this group. Thus it would appear that the *Nematostella *sequences in the R3 and R6 groups may have exchanged the FYVE domain. This may represent a novel, interesting genetic event in the evolution of the *Nematostella *myotubularin genes. Alternatively, it is conceivable that this might represent an error in genomic sequence assembly and annotation. Finally, the sequences M01745983C and Tad51955 are intriguing. These sequences cluster together consistently as a "new clade" in phylogenetic analysis based on alignments made from the PH-GRAM and phosphatase domains (see Legend to Table 2). In addition, each of them also possesses an N-terminal C2 domain (Protein kinase C **C**onserved region **2**; phospholipid binding), which has not been reported previously in Metazoan myotubularins. This data supports the existence of a previously undescribed myotubularin architecture, perhaps restricted to Choanoflagellates and early Metazoa.

It is clear from the above phylogenetic analysis that even in the genome of *Trichoplax*, the most deeply diverging Metazoan known [19], there is a representative in each of the six typical myotubularin similarity groups. This pattern is continued throughout the rest of the Metazoans. This indicates that the gene diversification into the three catalytically active and three inactive myotubularin groups had been completed at the very base of the Metazoan clade.

The situation is less clear for the *Monosiga *genome. Representatives can only be identified clearly for the R5, R6, and R9 groups. This would suggest that the split between catalytically active and inactive myotubularins characteristic of the Metazoan clade had occurred already in the common ancestor of Choanoflagellates and Metazoans. However, it is impossible to propose a precise model for this process, as three similarity groups have no identified members. This might represent a genuine absence, and therefore have evolutionary significance. On the other hand, it is conceivable that the apparent absence of myotubularin gene types is an artefact of genome assembly and annotation. That this might be the case is supported by the discovery of the partial sequence Mbrevi5R3, which was manually constructed from unassembled genomic sequence reads. It therefore seems most prudent to say that the precise status of myotubularin genes in Choanoflagellates will have to await the completion of genome sequencing projects for other species in this group.

#### Accessory Protein Domains

**Figure 6:**
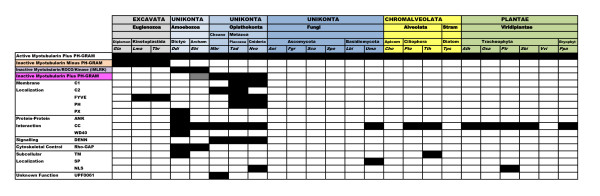
**Summary of Domain Distribution in Eukaryotic Myotubularin Homologues**. Myotubularin homologues were identified by HMM searches of protein datasets from completely sequenced eukaryotic genomes and domains were mapped as detailed in Methods. Colour code: In black, domain present; In white, domain absent; In gray, inactive myotubularin in *Entamoeba histolytica *plus PH-GRAM domain, relationship to similar proteins in Choanoflagellates and Metazoa indeterminate. IMLRK (inactive myotubularin/LRR/ROCO/Kinase proteins) of Amoebozoa also contain a PH-GRAM domain. Species are identified by a three letter abbreviation (**G**enus **sp**ecies [e.g. Gsp]). Species are as in Figure 1. Taxonomic descriptions and color coding are as in Figure 1. "Apicom" = Apicomplexa; "Archam" = Archamoebae"; "Bryophyt" = Bryophyta; "Choano" = Choanoflagellida; "Dictyo" = Dictyostelida; "Diplomon" = Diplomonadida; "Stramen" = Stramenopiles. Figure design after Gazave et al., 2009 [68].

Figure 6 presents a summary tabulation of the various domains found in myotubularin homologue sequences across the diverse eukaryotic groups examined in this study. It indicates the presence of active myotubularins with associated PH-GRAM domains in most species examined, across all the major supergroups. It summarizes the occurrence of the inactive myotubularins without PH-GRAM domains in the Excavates, the IMLRK proteins in the Amoebozoans, and the inactive myotubularins with PH-GRAM domains in the Choanoflagellates and Metazoa. This figure also indicates that several types of accessory domains are also sometimes observed in myotubularin homologues.

Nearly all animal myotubularins characterized to date possess coiled-coil domains C-term to the myotubularin domain. These have been shown to be important in mediating the protein-protein interactions between myotubularin subunits [3], and might conceivably provide interaction sites for other protein partners. We find that the presence of coiled-coil domains is much more sporadic in the entire myotubularin set (see Figure 6 and also Figure 2), with many proteins lacking them. Where they occur, the most common location is C-term to the myotubularin domain, however a number of sequences, particularly in the Amoebozoa, have N-term coiled-coil domains. The lack of coiled-coil domains in a number of myotubularins would suggest that potential protein-protein interactions would need to be facilitated by some other structural feature. It may be relevant in this context that PH domains, as a broad group, are known to often facilitate protein-protein interactions, as well as protein-lipid binding [20]. It may be that some of the structural and sequence diversity we observed in the PH-GRAM domains of the myotubularins in our sequence set arises due to this domain mediating protein-protein interactions. Protein-protein interactions might also be mediated by the observed ANK [**Ank**yrin], LRR [**L**eucine-**r**ich **r**epeat], and WD40 domains.

Several domains are found which typically mediate membrane localization (PH, PX [**P**ho**x**-like], C1, C2 [Protein kinase **C **conserved region **1 **and **2**], FYVE [**F**ab 1, **Y**O1B, **V**ac 1, and **E**EA1 (early endosome antigen 1)]), which is consistent with the postulated role of myotubularin proteins in vesicle transport. The presence of Rho-GAP domains might indicate a role in direct regulation of the cytoskeleton.

A few sequences show a predicted transmembrane domain, and one has a predicted signal peptide. These are very unusual for myotubularin sequences, and would be consistent with localization in a particular intracellular membrane compartment, and entry into the endomembrane system, respectively.

Finally, several sequences contain a predicted nuclear localization signal (NLS). This was true for Nv109357 (*Nematostella*), and several other metazoan members of the R5 clade. This data is presented in Additional File 2. Amongst these sequences was the *Drosophila *homologue of MTMR5/MTMR13. This is consistent with the observation that this protein (originally called "Sbf1" [SET binding factor 1]) co-localizes with the epigenetic regulatory protein Trithorax (Trx) on polytene chromosomes [21]. The presence in several members of the R5 clade of a well-conserved basic sequence loop and NLS prediction together suggest that nuclear localization may be possible for other members of this group. In addition, we observed predicted NLS in two sequences from the plant *Populus trichocarpa*. This data is summarized in Additional File 3. Recently the Arabidopsis myotubularin At3g10550 was shown to participate in a partially overlapping drought-response gene regulatory network with the epigenetic regulatory Trithorax homologue protein ATX1 [22]. This has raised the question as to whether this protein might be able to enter the nucleus. Our finding of a conserved basic sequence region supports this possibility.

### Myotubularin Gene Expression in Entamoeba

The unusually large complement of myotubularin homologues in *Entamoeba histolytica*, a well-known pathogenic organism, prompted us to explore the literature to examine patterns of myotubularin gene expression in this species. Davis et al. [23] reported differences in gene expression between the infective *E. histolytica *strain HM-1:IMSS and the non-pathogenic *E. histolytica *strain Rahman. Sequence EHI_141820 (one of the IMLRK proteins) showed an increase of 4.4× in expression (p = 1.07E-07). Ehrenkaufer et al. [24] identified "cyst-specific" *E. histolytica *genes which are differentially expressed in recent clinical isolates (which form cysts) as compared to laboratory strains or strains isolated from the mouse colon (which do not form cysts). Genes encoding two active myotubularins showed increases in expression (EHI_070120 [6.3×, p = 2.4E-03], EHI_049780 [5.3×, p = 7.3E-03]). Genes for four inactive myotubularins also showed increases in expression (EHI_140980 [6.3×, p = 5.0E-03], EHI_188050 [2.6×, p = 2.3E-03], EHI_185230 [10.7×, p = 7.4E-07], EHI_078170 [5.2×, p = 1.3E-04]). The latter two sequences are IMLRK proteins.

## Discussion

A variety of experiments in animal and fungal systems including in vitro enzymatic studies, mutational analysis, complementation assays, and in vivo overexpression, agree in characterizing myotubularins as phosphatases of the D3 position in the inositol headgroup of inositol phospholipids. PI3P and PI(3,5)P2 appear to be primarily localized to the cellular endomembrane system and restricted domains of the plasma membrane, mediating transitions between endosomes and lysosomes, retrograde transport between the endosomal compartment and trans Golgi network, and endocytosis of some materials from the cell surface [1,3]. Mutations of animal and yeast myotubularins lead to abnormal accumulations of PI3P and PI(3,5)P2, apparently disrupting normal cellular membrane trafficking events, perhaps through abnormal concentrations and/or localizations of PI-phosphate specific membrane-binding effector proteins [2-4]. One would anticipate that such intracellular membrane trafficking processes, and the mechanisms regulating them, would be very ancient, having arisen quite early in eukaryotic evolution. This is consistent with our most common observation of a small number of myotubularin genes in organisms across a broad phylogenetic distribution, suggesting the presence of a single such gene in the last common ancestor for all extant eukaryotic groups. The PH-GRAM domain appears to be a very early acquisition, perhaps coincident with the divergence of a generic PTP domain into the characteristic elaborated myotubularin phosphatase domain.

Inactive myotubularin subunits are one of the particularly interesting features of this gene group. Our data are consistent with these having appeared on three separate occasions in eukaryotic evolution, in different taxonomic groups. The distinctive lack of a PH-GRAM domain in the inactive Excavate myotubularins makes it likely that these represent a unique lineage. Similarly, the IMLRK domain architecture of the Amoebozoa inactive myotubularins suggests they too have a unique origin. Finally, it is likely that an active myotubularin lineage then began an independent diversification event somewhere around the base of the Choanoflagellate/Metazoan divergence to produce the six similarity groups characteristic of the Metazoans. This is consistent with our finding of all six myotubularin subgroups being identifiable in the deeply diverging Placozoan *Trichoplax*, but only three subgroup representatives being clearly identifiable from the Choanoflagellate *Monosiga*. More completed genome sequences from Choanoflagellates and even more deeply diverging protistan "animal allies" (e.g. Ichthyosporea and Filasterea [25,26]) will be necessary to precisely define this pivotal period in myotubularin gene history.

Myotubularin function has been most intensively studied in humans, where a number of diseases arising from inherited mutations have been characterized. It has been suggested that a common unifying pathophysiological mechanism in these disorders may be abnormality in the membrane trafficking necessary to alter the characteristic molecular composition and identity of the plasma membrane and specialized derivative membrane structures during cellular differentiation [4]. In this model the disordered membrane trafficking would be secondary to perturbations in the normal levels and perhaps subcellular distribution of PI3P and PI(3,5)P2, the normal substrates of myotubularins. This model suggests that the normal function of myotubularins becomes especially critical in situations where cells are required to turn over and alter, on a large scale, through membrane trafficking, the suites of proteins and perhaps lipids characterizing particular domains on the plasma membrane and components of the endomembrane system.

Myotubularin genes have undergone an expansion in the Amoebozoan species *Dictyostelium discoideum*. Nine myotubularins are predicted to be enzymatically active, and two inactive. Nothing is known about the function of the myotubularins in this organism. However, it is reasonable to suggest that they are involved in the regulation of the substantial intracellular trafficking events that would accompany membrane reorganization during a complex life cycle. The two inactive *Dictyostelium *myotubularins also possess the distinctive IMLRK domain architecture. "ROCO" proteins (which usually contain LRR/ROCO/kinase domains, but not myotubularin domains) were initially characterized in *Dictyostelium*, are biochemically best understood in this organism, but have a widespread phylogenetic distribution in both prokaryotes and eukaryotes [13]. In *Dictyostelium*, where there are 11 ROCO genes in all, functional evidence is available for four: gene gbpC is involved in chemotaxis; genes QkgA/roco2 and roco5 are involved in growth and development; and gene pats1 (our IMLRK sequence DDB0191503) is involved in cytokinesis. The ROCO proteins have recently received considerable attention because in humans the family member LRRK2 is involved in familial and some cases of sporadic Parkinson's disease [13]. Biochemical approaches, analysis of disease-associated mutations, and solved protein structures have revealed that the protein kinase domain is regulated by the GTPase activity of the ROC domain, through protein-protein dimerization mediated by the COR domain [15,27]. Thus these proteins have been likened to a "stand-alone" intramolecular signal transduction cascade, mediated by their multiple functional domains. *Dictyostelium *pats1 (DDB0191503) is essential for cytokinesis, and contains an enzymatically inactive myotubularin domain, whose function has not been experimentally tested. A reasonable proposal would be that the myotubularin-like portion of the protein could provide membrane localization via its PH-GRAM domain. It is known that specialized plasma membrane domains enriched in PI(4,5)P2 accumulate at the intercellular bridge during cytokinesis, where they regulate the underlying actin cytoskeleton [28]. The *Dictyostelium *gene roco9 (DDB0191512) also encodes an IMLRK protein. Nothing is known about the function of this protein, but it contains a Rho-GAP domain, which might indicate a role in regulation of the actin cytoskeleton. Once again, the myotubularin-like region of the protein could supply membrane localization. Another functional possibility for the inactive myotubularin domains of both pats1 and roco9 is that they might bind to one or more of the many active *Dictyostelium *myotubularins, and mediate regulation of their activities. Several such combinations of active plus regulatory inactive myotubularin subunits are well characterized in animal cells [3].

In *Entamoeba*, another Amoebozoan, there is an even larger myotubularin gene set than observed in *Dictyostelium *- there are 8 active myotubularins, and 11 inactive myotubularins (9 of them with the IMLRK domain architecture). This is the largest collection of myotubularin genes observed to date in any eukaryotic genome examined. This large repertoire of active plus inactive subunits suggests the possibility of a particularly rich network of regulatory protein-protein associations. It is particularly striking that, in contrast to the intricate multicellular associations of *Dictyostelium*, the *Entamoeba *life cycle is morphologically rather simple. Underlying this apparently simplicity, however, is probably complex turnover and change to plasma membrane protein sets accompanying life cycle transitions and invasive contact with host tissues [29-31]. It might be hypothesized that the large complement of myotubularin genes found in this organism is necessary for precise spatial and temporal regulation of these membrane trafficking events, over and above the "constitutive" requirements of any eukaryotic cell. Their numbers would suggest that the IMLRK proteins might be particularly important. The data suggest that the protein kinase domains of the IMLRK proteins will be active, and that the ROC domains lack GTPase activity. This would indicate a change to the typical paradigm of ROC GTPase-mediated control of the kinase domain. It is possible that the divergent ROCO domains in these proteins effect protein kinase regulation via interaction with novel accessory proteins.

In most human cases of infection with *Entamoeba histolytica*, the organism remains in the lumen of the intestine, in contact with the epithelium. In a minority of cases, invasion of the intestinal wall occurs, which may lead to liver abscesses. The life cycle is completed by the organism forming cysts, which are released from the host in excrement, to infect new hosts. A significant increase in gene expression was noted in a myotubularin gene in a pathogenic vs a non-pathogenic strain of *E. histolytica *[23]. Significant upregulation was noted in several myotubularin genes which appear to be acting specifically in the encystment stage of the life cycle [24]. Taken together, these data suggest that myotubularin genes are important to both completion of the life cycle, and invasive disease in this organism.

## Conclusions

We have presented a phylogenetic survey of myotubularin genes across a diverse array of eukaryotes, including distribution, domain architecture, and inferred evolutionary history. We have characterized an expansion of genes in the Amoebozoa encoding proteins with the novel combination of "IMLRK" (inactive myotubularin/LRR/ROCO/kinase) domains. This group is particularly prominent in the pathogenic organism *Entamoeba histolytica*, which contains the largest myotubularin gene family of any eukaryotic genome yet examined. Gene expression data in *E. histolytica *indicates that myotubularin function may be important to both critical life cycle transitions and host infection. The data indicate that pathogen myotubularin genes may be important targets for basic research, and perhaps novel strategies for disease control.

## Methods

### Identification of Putative Myotubularin Homologue Sequences

Sequences of all 14 human myotubularin proteins were obtained from NCBI Entrez [32]. A multiple sequence alignment was constructed and edited as presented in the next section. Eukaryotes with a completely sequenced genome were identified using the Genomes Online Database [33,34], and organismal protein datafiles were obtained from the sites linked therein. A Hidden Markov Model (HMM) of the human myotubularin multiple sequence alignment was constructed using the HMMER program package, which was then used to search the various eukaryotic protein sequence datafiles (program commands "hmmbuild", "hmmcalibrate" and "hmmsearch", threshold E = 1). Candidate sequences were determined by a combination of low E value (generally less than E = 0.01) and a long alignment to the HMM model. A spreadsheet with the URLs of websites used to obtain protein datasets within which candidate myotubularin homologue sequences were found is presented in Additional File 4.

### Determination of Myotubularin Similarity Regions within Sequences

Candidate myotubularin sequences obtained from the initial HMM search of protein datafiles were subjected to sequence:profile (FFAS03) [35,36] and profile:profile (HHPred) [37-39] analysis to identify the boundaries of the characteristic PH-GRAM and myotubularin phosphatase domains, by comparison with the solved structures of human MTMR2 (PDB: 1LW3, 1ZSQ). For most sequences this was a contiguous region, which was then utilized for multiple sequence alignment. FFAS03 returns standardized variable ("Z") scores for comparisons between a query and a solved template structure sequence, with a score of 9.5 cited by the authors as being statistically significant. Candidate myotubularin sequences routinely exceeded this threshold. HHPred returns a probability score reflecting both the alignment between HMMs formed based on the query sequence and solved structure sequences, and predicted secondary structure. A probability of 95% is cited by the authors as having a very low false positive rate. Candidate myotubularin sequences routinely exceeded this threshold.

### Characterization of Non-Myotubularin Domains within Sequences

Candidate myotubularin homologue sequences obtained by HMM search as described above were examined for functional domains using FFAS03 and HHPred as described above (except now using as a comparison all sequences with solved structures in the PDB), and also NCBI CDD [40,41], Pfam [42,43], and InterProScan [44,45], all with default settings. For the identification of ROCO domain sequences the comparison structure was that of the ROCO domain of *Chlorobium tepidum *(PDB: 3dpu_A [15]). The identity of the domains was confirmed by successive rounds of multiple sequence alignment (as detailed below), Hidden Markov Model construction (as detailed above), and database searching.

### Characterization of Additional Protein Sequence Features

Candidate myotubularin homologue sequences obtained by HMM search as described above were examined for the presence of predicted signal peptides (Phobius [46,47], SignalP [48,49]), predicted transmembrane helices (Phobius [46,47], TMHMM [50,51]), predicted coiled-coil regions (Marcoil [52,53], PairCoil2 [54,55]), and nuclear localization signals NLStradamus [56,57]).

### Multiple Sequence Alignment

#### Candidate Myotubularin Sequences

Candidate myotubularin sequences (including both the PH-GRAM domain and the myotubularin phosphatase domain, or just the phosphatase domain alone (as defined by the sequence of the solved structure of MTMR2_Hu (PDB:1LW3)) were aligned utilizing as necessary several multiple sequence alignment programs: Muscle [58], T-Coffee [59] or M-Coffee [60,61]. Quality of alignments was guided by evaluation at the T-Coffee web server. In some instances, sub-alignments were constructed, and then either sequences, or other sub-alignments were added using the Profile alignment mode of T-Coffee or ClustalX [62] (default program settings). Alignments were displayed and edited using the program GeneDoc [63]. After alignment analysis, it was found that some database sequences for candidate myotubularin homologues were incomplete due to annotation mistakes. These were supplemented with additional sequence by use of the appropriate organismal genome browser, and search of the organismal genomic DNA utilizing TBLASTN. Such sequences are denoted with the suffix "C" in the figure legends. For the reference multiple sequence alignment presented as Additional File 1 (100 sequences), no sequence regions were deleted.

#### Protein Kinase and ROCO Domains

Protein kinase domain and ROCO domain sequences within some myotubularin homologue candidates, detected as described above, were subjected to multiple sequence alignment with M-Coffee, displayed and edited with GeneDoc, as described above.

### Phylogenetic Tree Inference

Multiple sequence alignments were constructed as detailed above. In some instances rapidly evolving sites (Category 8) were identified with PAML [64] and removed from the alignment (analysis performed using the programs AIR-Identifier and AIR-Remover at the University of Oslo BioPortal http://www.bioportal.uio.no/.

Bayesian phylogenetic trees were inferred with PhyloBayes 3.2d [65]. Two independent Markov Chains were run under various amino acid substitution models, and between-sites rate variation models (UL3, Dirichlet; UL3, Uniform; WLSR5, Dirichlet) for approximately 5,000 cycles, using a 20% (approximately 1,000 cycle) burn-in. Chain convergence was checked using the statistics "maxdiff" < 0.10 and "effsize" > 100. Maximum likelihood trees were inferred with PhyML 3.0 [66] and PhyML-mixture [67]. A two-stage process was used [6], where first the best tree was inferred from 20 random starts, using SPR moves, from a Parsimony input tree (PhyML) or a BioNJ input tree (PhyML-mixture). Various amino acid substitution models and models for between site rate variation were used ([JTT plus 4 Gamma categories, empirical amino acid frequencies, proportion of invariant sites estimated], [WAG plus 4 Gamma categories, empirical amino acid frequencies, proportion of invariant sites estimated], [LG plus 4 Gamma categories, empirical amino acid frequencies, proportion of invariant sites estimated], [EX3, single rate category, model amino acid frequencies]). Then a second stage utilized the best tree from the first stage as a user input tree, and inferred 100 bootstrap replicates, using SPR moves, employing the same amino acid substitution and site rate variation parameters as in the first stage.

## Abbreviations

ANK: Ankyrin domain; CDD: Conserved Domain Database; CMT: Charcot-Marie-Tooth; C1: Protein kinase C conserved region 1 (C1) domain (Cysteine-rich domain); C2: Protein kinase C conserved region 2 (C2) domain (Cysteine-rich domain); DENN: differentially expressed in neoplastic versus normal cells; FFAS: Fold and Function Assignment System; FYVE: Fab 1, YO1B, Vac 1, and EEA1 (early endosome antigen 1); GAP: GTPase activating protein; GRAM: glucosyltransferases, Rab-like GTPase activators and myotubularins; HHPred: HMM-HMM structure prediction; HMM: Hidden Markov Model; IMLRK: Inactive myotubularin/LRR/ROCO/Kinase domain architecture of Amoebozoa; LRR: Leucine-rich repeat; MTMR: MTM1-related; NLS: Nuclear localization signal; PDB: Protein Data Bank; PH: Pleckstrin-homology; Pfam: Protein Families; PKA: Protein kinase A; PTP: Protein tyrosine phosphatase; PX: Phox-like; Rho-GAP: Rho-GTPase Activating Protein; ROCO: Ras of complex proteins (ROC) + C-term of ROC (COR); TKL: Tyrosine kinase-like; WD40: structural motif of 40-43 amino acids in the beta subunit of G-proteins; XLMTM: X-linked myotubular myopathy

## Authors' contributions

GBGM and DK conceived of the study. DK designed the implementation of the study. DK collected all sequence data, performed domain mapping, multiple sequence alignment, phylogenetic tree analysis, and mined published gene expression data. DK composed the data figures and tables. DK and GBGM wrote and approve the manuscript.

## Supplementary Material

Additional file 1 **Full Myotubularin Sequences Alignment**. This file presents the full myotubularin sequences alignment, a portion of which was presented in Figure 3. All details of this alignment are the same as described in the Legend to Figure 3, except that in this figure a blue bar is used to denote the extent of the N-terminal PH-GRAM domain, and an orange bar denotes the extent of the phosphatase domain catalytic signature motif. Click here for file

Additional file 2 **Predicted Nuclear Localization Signals (NLS) in Animal Myotubularin Homologue Sequences**. This file presents data summarizing predicted nuclear localization signals (NLS) in metazoan myotubularin homologue sequences of the R5 clade. Click here for file

Additional file 3 **Predicted Nuclear Localization Signals (NLS) in Plant Myotubularin Homologue Sequences**. This file presents data summarizing predicted nuclear localization signals (NLS) in plant myotubularin homologue sequences. Click here for file

Additional file 4 **URLs for Protein Databases**. This file contains the URLs for downloading of all organismal protein datasets searched for myotubularin homologues in this study. It also contains the original literature citation for the publication of each completely sequenced organismal genome. Click here for file

Additional file 5 **Myotubularin Protein Sequences**. This file contains the FASTA-formatted sequences for all myotubularin homologues identified in this study, reference human myotubularin proteins, database accession numbers, and sequence designations as used in the data figures. Click here for file

Additional file 6 **Additional Information on ROCO Sequence Alignment**. This file presents species designations and database accession numbers for sequences presented in the multiple sequence alignment of Figure 4. Click here for file
