# Supplementary material for: A phylogenetic survey of myotubularin genes of eukaryotes: distribution, protein structure, evolution, and gene expression
Source: BMC Evol Biol. 2010 Jun 24;10:196. doi: 10.1186/1471-2148-10-196 (PMC2927912; doi:10.1186/1471-2148-10-196)
Supplement: Additional file 1 — Full Myotubularin Sequences Alignment. This file presents the full myotubularin sequences alignment, a portion of which was presented in Figure 3. All details of this alignment are the same as described in the Legend to Figure 3, except that in this figure a blue bar is used to denote the extent of the N-terminal PH-GRAM domain, and an orange bar denotes the extent of the phosphatase domain catalytic signature motif. [file 1471-2148-10-196-S1.PDF]

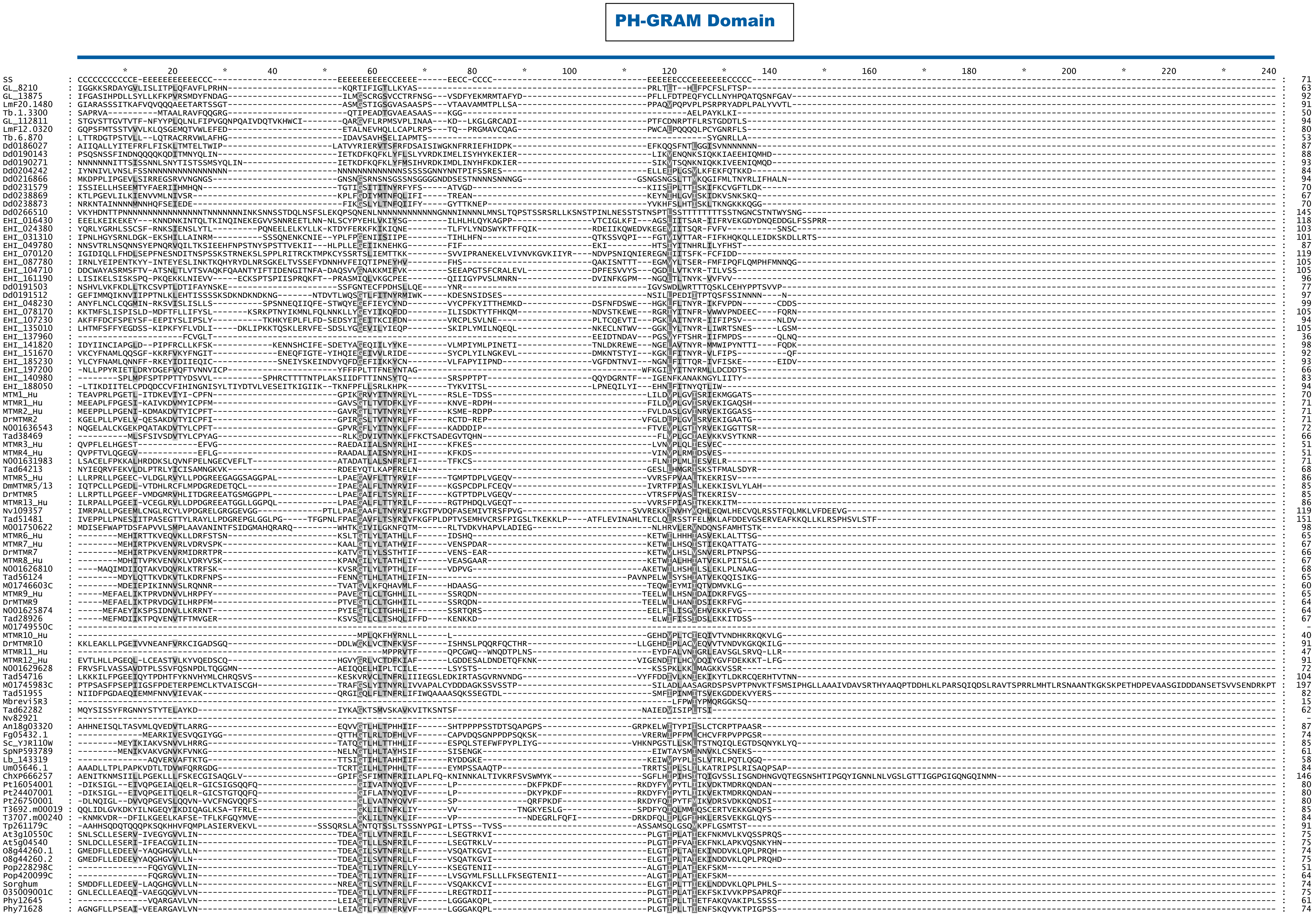

PH-GRAM Domain

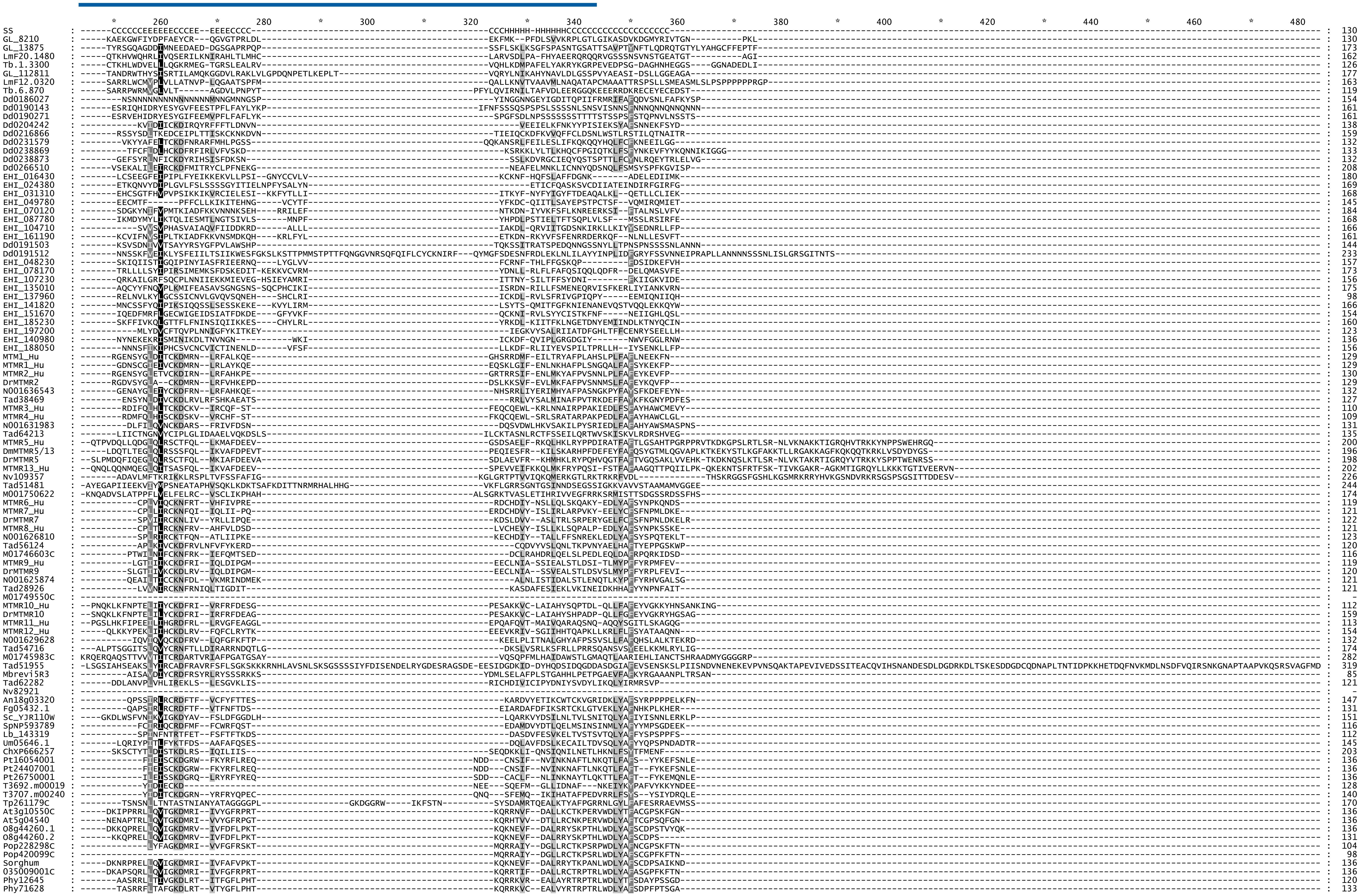

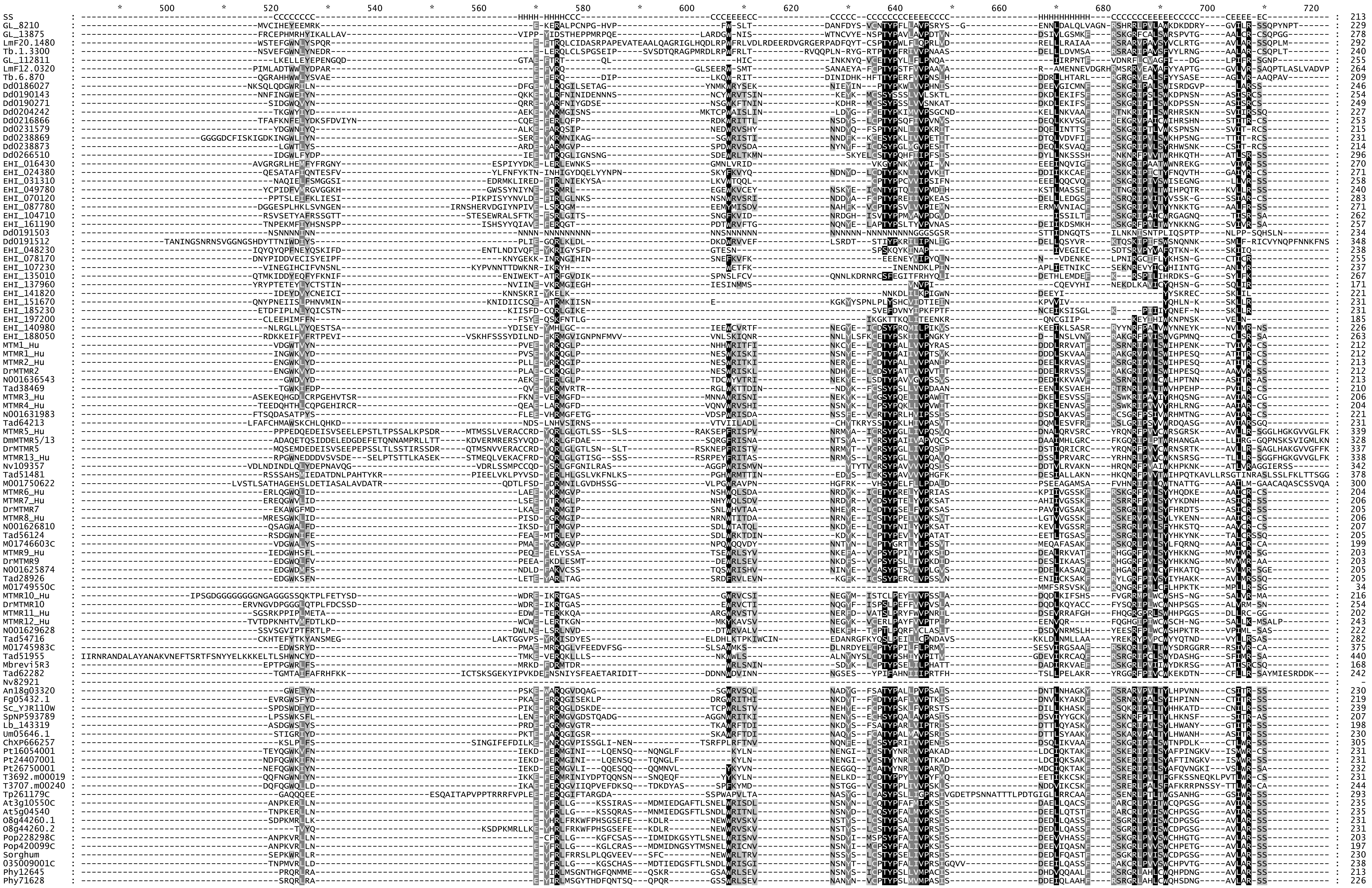

```
                              *     740         *       760         *       780         *       800         *       820         *       840         *       860         *       880         *       900         *       920         *       940         *       960
SS            : ------------------------CCCCC--------CCCCCCHHHHHHHHHHHHCCCCCCCC---------------------------------------------------------------------------------------------------EEEEECCCHHHHHH---HHHCCC-C-----------------------------------------CCCCC---CCC------------------------- : 271
GL_8210       : ------------------------------K--------KSDSKFVADRAYLQHIWQ-HYGALHKKE--------------------------------------------------------------------------------------------KLLVIDARTRANMQ---MNQFVG-R----------------------------------------GTE-----GYPFVHVEFLDIPGCQYIQ------ : 300
GL_13875      : ------------------------------G--------FGLSRSSDDEALLRAVCN-APPSTDTRN-----------------------------------------------------------------------------------------------LLFVFDARPHLAAF---ANRLTGK-----------------------------------------GFEST--RAYPF--------------------- : 337
LmF20.1480    : ------------------------------------------RSPQLSADSDVCYTLINMGYAPHHARPQRTTASAAVSSSL-------------------------------------------------------------------------VPSLLATAHGSSPAA---TNSLKASP---VVGAPAAVARPVRPPSLFDDDSDGDGQPPCAGST--SNASSTQQL------------------ : 398
Tb.1.3300     : ------------------------------------------RSSLMRADGELCSMLTN-DYTEHNAAP-------------------------------------------------------------------------------------------NQDMSKTRAAAAAV---VETVAQTS--------------TKPPPPLFDTDVEEGKTSGKETC--GSSPVNQQQ------------------ : 320
GL_112811     : -------------------------------------ILGIGANPIADFLD-FYMPSTCYK--------------------------------------------------------------------------------------------------RAIVFSLGPTTYYD---IQKPVSTM-------PPN--------------------SNGTFI--DYANYLRVLQ------------------ : 319
LmF12.0320    : TEQALEWTSRMTGGRLGRAAPAASSSQT-------QTSSLVVPLRDVLRLTLS-AFAAAAGVTAQ-------------------------------------------------------------------------------------------RIFVVDLRSARAAF---ANVGKGGGTMELLLYPFG---------------------------GVEYA--GLPNIHSVQKAWRRLRASLTQDAPY : 383
Tb.6.870      : ------------------------------I--------PALYASAASAKALTDPVE-EYRNACMRP-------------------------------------------------------------------------------------------VHIFDLRSGIRAL---SSTLVGGG-----FSLG--------------------ENRRFC--SLENIHHVRESYD---------------- : 281
Dd0186027     : -------------------------QPMVG-------ITRQKSVVDEKLVDSIRMACPSGKS---------------------------------------------------------------------------------------------------LFLLDARPKANAI---ANVAKG-----------------------------------------MGYELN----------------------------- : 301
Dd0190143     : -------------------------QPLVG-------IERSRCPEDELYCSNALSAPSKT----------------------------------------------------------------------------------------------------LYLIDARPKLNAY---ANTANG-----------------------------------------ACFENI--HN------------------------- : 310
Dd0190271     : -------------------------QPLVG-------IERARCPEDEFYCSSALSSPSKT----------------------------------------------------------------------------------------------------LYLIDARPKINAI---ANTANG-----------------------------------------ACFENI--HN------------------------- : 305
Dd0204242     : --------------------------PKVG-------LQRTHSKDDEYLINMFRIAANQMNP--------------------------------------------------------------------------------------------------LVVADARPKMNAV---ANQAKG-----------------------------------------ACYEDI--QR------------------------- : 283
Dd0216866     : -------------------------QPRVG-------LARARCSEDEEYLNIITKSTNYTTNSP----------------------------------------------------------------------------------------------ILYVMDSRPMANAK---ANALLG-----------------------------------------KCHENT--S-------------------------- : 312
Dd0231579     : -------------------------QPVIG-------VTKKRSLDDEKLLKESILLANPQSEK-------------------------------------------------------------------------------------------------VYIMDARPQLNAV---ANQMMG-----------------------------------------LCYEDT--NNQS----------------------- : 274
Dd0238869     : -------------------------QPLVG-------LTGSRCIDDELLIKQISSPTIIQQPQSQQQSSSPPPSFINYSSTTSTNSTPNLNQINNINDNFKSLNNSGNSIVFNKRQSYISNSLSNLESSLVSNNNNNNSNINNNNNNNNVEKILNLLDARPKVNAM---SNKAFMG---------------------------------------ACFENV--STF------------------------ : 381
Dd0238873     : -------------------------QPLVG-------IGGNRSTEDENLLNSIRMTSTAGSGSSGGSGGSGSN-------------------------------------------------------------KENNSSDRTLYLILDCRPYANAV---GNRAKG-----------------------------------------ACWEIL--NNY------------------------ : 294
Dd0266510     : -------------------------Q-QTG-----------KSRCEEDELLIQAIRKSKTILPNNNNQQQQPQQQQQ--------------------------------------------------------------------------------TLYHIDIKSTSSSP---TSSSSS-----------------------------------------HCHEDI--S-------------------------- : 362
EHI_016430    : -------------------------QPLVG----L----KTSRCFSDEKLLEKA----AQLSKNKR------------------------------------------------------------------------------------------ISHIIDCRPKLNAS---ANL-IKGM----------------------------------------GFEDI--RYY------------------------ : 327
EHI_024380    : -------------------------QPLVANSSYFIGVIKDSVSKEDEEFLSEI----RTIGKNDDAI------------------------------------------------------------------------------------------LYIFDCRPVTNAY---ANR-VNGG----------------------------------------GYENT--NAY------------------------ : 338
EHI_031310    : -------------------------QPATG---I----TSSRSKVDEQMLTEY----AMMSRTHS-------------------------------------------------------------------------------------------IKVYDARPKLNAN---VNR-VKGM----------------------------------------GSENM--KNY------------------------ : 315
EHI_049780    : -------------------------QPKTG--------FTGKSDEDVTLLYR------EGGGSY----------------------------------------------------------------------------------------------IQIYDCRDYYSAL---GNKAFKQA----------------------------------------GFESI--SEY------------------------ : 294
EHI_070120    : -------------------------QPLCG----L----TGRKCQADIELLKMI----THGGPSHK------------------------------------------------------------------------------------------LRILDSRPKANAV---ANI-AMGG----------------------------------------GYETE--EDY------------------------ : 340
EHI_087780    : -------------------------QPLVA-TTFAFGVFKKSESIDDQNYITHI----RCDGRDDKSV----------------------------------------------------------------------------------------LIVLDCRAMANAV---ANR-LSGG----------------------------------------GYETS--GSY------------------------ : 337
EHI_104710    : -------------------------QPLPG-----A----LGRARCEADELLLKTL----VTLTKGGEI------------------------------------------------------------------------------------------LSIFDARPPLNAQ---ANR-LQGG----------------------------------------GVESN--TYY------------------------ : 321
EHI_161190    : -------------------------QPLPG---MVTPVTGKNTSGDVTYLQLI----DETTGKRG--------------------------------------------------------------------------------------------IHVLDSRPKLNAY---TNK-AAGG----------------------------------------GYEDE--TTY------------------------ : 318
Dd0191503     : -------------------------LLMSP-------QTSSPRIHQSISSSSIPQVCQEDIDFL----------------------------------------------------------------------------------------------RGILDIKSSS-MLNVFDTG-SGGSY-------------------------------------SSTM------------------------------ : 293
Dd0191512     : GGTLAYNNNSTIINNNSVIITNSSTIGT-------NTSGISNSNHLIVGEIEPPSPIKKRQ-------------------------------------------------------------------------------------------------QQIVDKKDTSSPLSSLRSS-KGIPS-------------------------------------KSSKKDKQGFLSSSTSSIIPIQTTISTDTV--- : 456
EHI_048230    : ----------------------------------------------FSLSSSISLNS--------------------------------------------------------------------------------------------------------PTPIIFNSSISNSV---VKYLI---------------------------------------------------------------------- : 267
EHI_078170    : ------------------------------------LSLSKTVDKTKSFFL---------------------------------------------------------------------------------------------------------------STIDEVQKKYHAI---YLS------------------------------------------------------------------------ : 286
EHI_107230    : -------------------------------------------------LTI------------------------------------------------------------------------------------------------------------------PFIKNLKNEITID---IIND----------------------------------------------------------------------- : 257
EHI_135010    : ------------------------------------LQYIFCKDHTTSQLK---------------------------------------------------------------------------------------------------------------YPDELKQIFTTI---CTTEIVFVN------------------------------------------------------------------ : 303
EHI_137960    : --------------------------------------------------FPLLKN----------------------------------------------------------------------------------------------------------VYADTVLSSFTIK---MKEG----------------------------------------------------------------------- : 194
EHI_141820    : -----------------------------------------IDESEKGGRIFR---------------------------------------------------------------------------------------------------------------IEINNDKNEINNK---RISENIQG------------------------------------------------------------------- : 254
EHI_151670    : -------------------------------------------------LAK------------------------------------------------------------------------------------------------------------------IINEDLYEEVYEI---GSKAE---------------------------------------------------------------------- : 252
EHI_185230    : -------------------------------------------------FP-------------------------------------------------------------------------------------------------------------------IGINDTNESINTT---TFT-LYES-------------------------------------GITILP----------------------------- : 259
EHI_197200    : ------------------------------------ICQTGMTTNNNYIQVNGPDIPFAS----------------------------------------------------------------------------------------------------LIELINNNIGTPIS----DDIFN------------------------------------------------------------------- : 228
EHI_140980    : -------------------------ATIKG-------------CYNDTQIPFA-----FTNKKN--------------------------------------------------------------------------------------------------LVIMDFVDKITSC---KML-------------------------------------------------------L--------------- : 264
EHI_188050    : -------------------------QPLPG-----T---FGQIKCESDVSILQSM-----MELCGKKT-----------------------------------------------------------------------------------------FFMYDARPKLNTK---ATR-FQGG----------------------------------------GMESQ--STY------------------------ : 321
MTM1_Hu       : -------------------------QPLVG-------MSGKRNKDDEKYLDVIRETNKQISK-------------------------------------------------------------------------------------------------LTIYDARPSVNAV---ANKATG-G-----------------------------------------GYESD--DAY------------------------ : 270
MTMR1_Hu      : -------------------------QPLVG-------PNDKRCKEDEKYLQTIMDANAQSHK--------------------------------------------------------------------------------------------------LIIFDARQNSVAD---TNKTKG-G-----------------------------------------GYESE--SAY------------------------ : 270
MTMR2_Hu      : -------------------------QPMVG-------VSGKRSKEDEKYLQAIMDSNAQSHK--------------------------------------------------------------------------------------------------LFIFDARPSVNAV---ANKAKG-G-----------------------------------------GYESE--DAY------------------------ : 271
DrMTMR2       : -------------------------QPMVG-------QNGRRCKEDEKLLQAIMDANAQSHK--------------------------------------------------------------------------------------------------LFIFDARPSVNAA---ANKMKG-G-----------------------------------------GYESE--DAY------------------------ : 270
N001636543    : ---------------------------QPNR-------GLLARNKDDEKYFQAIMKANPQCHK--------------------------------------------------------------------------------------------------LLIFDARPRINAV---ANQAKG-G-----------------------------------------GYETE--ENY------------------------ : 270
Tad38469      : -------------------------QPTSG-------LIDKPSEEDKNYFRLIWEANSDQK----------------------------------------------------------------------------------------------------IYVVDARPKKNAV---ANKLKG-G-----------------------------------------GFEAN-------------------------- : 264
MTMR3_Hu      : -------------------------QPEVS-------WWGWRNADDEHLVQSVAKACASD--SRSSGSKLSTRNTSRDFPNGGDLSDVEFDSSLSNAS-------------------------------GAESLAIQPQKLLILDARSYAAAV---ANRAKG-G-----------------------------------------GCECP--EYY------------------------ : 309
MTMR4_Hu      : -------------------------QPEIS-------WWGWRNADDEYLVTSIAKACALDPGTRATGGSLSTGNN-----DTSEACDADFDSSLTACS---------------------------------GVESTA-APQKLLILDARSYTAAV---ANRAKG-G-----------------------------------------GCECE--EYY------------------------ : 303
N001631983    : -------------------------QPQVG-------WLGWRSAEDEILLQAIGQACTSDRYPSVAKLLNGITVNGINKKHDMGDVLNGPADMSEDGP-----------------------------------------IARNPKQVLIVDARSYTAAV---ANRGKG-G-----------------------------------------GYEYT--EYY------------------------ : 322
Tad64213      : ---------------------------PQVA-------FLGYRCAEDESLIRAIAESSTGDKVNEDELTN-------------------------------------------------------------------------------------GMVASACAGSLEDKSFYNNFSPFN----------------------------------MSASLN---------------------------- : 297
MTMR5_Hu      : AQNAP--------------------SPGQS-------QADSSSLEQEKYLQAVVSSMPRYADASGRNTLSGFSSAHMGSHGKWGSVRTSGRSSGLGT---DVGSRL-------------------AGRDALAPPQANGGPPDPGFLRPQRAALYIL---GDKAQL-K-----------------------------------------GVRSD--PL------------------------- : 460
DmMTMR5/13    : TTGS---------------------TTNAH-------HDVTHYPEQDKYFLALINTMPKLTPLALNQYSGMNLSMSSLMGHSSSDDRQPLTPELSRKHKNNLDISD-----------------GNKSSQGGKGGTMKGNPKNSLAHPFRKMRLY---ALGEKS-Q-----------------------------------------AKSNM--NVD------------------------ : 452
DrMTMR5       : SPNAP--------------------TAGPS-------QADSTSLEQEKYLQAIISSMPSYSETSGRNTLSGFTSSHMSTSGKWGSIRGSGRLSTYNPDVG-----------------------NRLAGKESPQANGGPSETYFLRQQRAYLFII---GDKSQL-K-----------------------------------------GGKQD--SF------------------------- : 455
MTMR13_Hu     : SQNSPQA------------------APTSS-------LESSSSIEQEKYLQAILNAVSVHQKLRGNSTLT-VRPAFALSPGVWASLRSSTRLISSPTSFIDVGARL-----------------AGKDHSASFSNSSYLQ--NQLLKRQAALYIF---GEKSQL-K-----------------------------------------NFKVE--FA------------------------- : 461
Nv109357      : -----------------VASMIRSGMGVGGSMSGNSIPSSSVEQEKLFAAVAKAITIYWVFCIRSQS--------------------------------------------------------------------------CCFPAVSATPLSKNKSVARQNSLHHGVILN-------------------------TEGYFNQ--DKRDETDRGQLVEATLKKKGWEPAA : 454
Tad51481      : S------------------------EGNAG----VSSGTTADNEAEEEKYLTAIITGTPRIPKLKTGKSPNPAKRRNSSSSPRSNRNARIGRTASRKVTQIQSQTIKRLTTPNSSPH----------RDPVSENNDGYLNNTMYADSLIDKRLAGKDW---EAAHLYVL----------------------------------------GDKS------------------------------ : 510
M001750622    : FYRALLD------------------LRVKP-------VRGQNAQGSQRLMDLKLKPVSKG--------------------------------------------------------------------------------------------------SQAQPQIVVL---GDNAST-Q-----------------------------------------DPQIP--GV------------------------- : 361
MTMR6_Hu      : -------------------------QPLSG-------FS-ARCLEDEHLLQAISKANPVNRY------------------------------------------------------------------------------------------------MYVVDTRPKLNAM---ANRAAG-K-----------------------------------------GYENE--DNY------------------------ : 261
MTMR7_Hu      : -------------------------QPLSG-------FS-ARCLEDEQMLQAIRKANPGSDF------------------------------------------------------------------------------------------------VYVVDTRPKLNAM---ANRAAG-K-----------------------------------------GYENE--DNY------------------------ : 263
DrMTMR7       : -------------------------QPLSG-------FS-ARSSDDEQMLQAIMKSNPVSEF------------------------------------------------------------------------------------------------MYVVDTRPKLNAM---ANRAAG-K-----------------------------------------GYENE--DHY------------------------ : 262
MTMR8_Hu      : -------------------------QPLSG-------FY-TRCVDDELLLEAISQTNPGSQF------------------------------------------------------------------------------------------------MYVVDTRPKLNAM---ANRAAG-K-----------------------------------------GYENE--DNY------------------------ : 263
N001626810    : ---------------------------QPLG-------GFKGRCPEDEQMIQALLNSNPNSKF-----------------------------------------------------------------------------------------------IYIVDTRPRLNAL---ANRAAG-K-----------------------------------------GYESE--DFY------------------------ : 264
Tad56124      : --------------------------PLTG-------MRGRSVDDEQLLQAVIQSNPKSP-----------------------------------------------------------------------------------------------VMYIVDTRPKINAM---ANKAAG-K-----------------------------------------GYENL--DNY------------------------ : 261
M01746603C    : -------------------------QPLTG-------VQAKRSEDDEALLEFIRLCNPHSEL-----------------------------------------------------------------------------------------------PLIIVDTRPQMNAM---ANKAAG-K-----------------------------------------GFENM--EGY------------------------ : 258
MTMR9_Hu      : -------------------------QPLTG-------TNGRRCKEDEKLINATLRAGKR----------------------------------------------------------------------------------------------------GYIIDTRSLNVAQ---QTRAKG-G-----------------------------------------GFEQE--AHY------------------------ : 258
DrMTMR9       : -------------------------QPLTG-------TNGRRCKEDEKLINATLCAGKR----------------------------------------------------------------------------------------------------GYIIDTRTIAVAQ---QAKARG-G-----------------------------------------GFEQE--ANY------------------------ : 258
N001625874    : -------------------------PLCGS-------MSNKRCKEDEKLVNSALGSMKKG-------------------------------------------------------------------------------------------------------MIVVDTRSTPAVM---NSRSKG-G-----------------------------------------GLPE--SNY------------------------ : 260
Tad28926      : --------------------------PLSG-------PNELRCKEDEKLINAAIFGPKH-----------------------------------------------------------------------------------------------------CYIVVDTRLTNYATN-IKNKGGGVEPS-------------------------------------------------------------------- : 256
M01749550C    : -------------------------QPLAG-------SSGKRKYEDEQMLRMYLSTADVKQG-------------------------------------------------------------------------------------------------LILLDTRTSSVAS---THRSKG-G-----------------------------------------GLEVI--DKY------------------------ : 91
MTMR10_Hu     : ----------------------------LIK-------DVLQQRKIDDRICNAIITKSHPQR-----------------------------------------------------------------------------------------------------------SDVYKSD--LDKT-------------------------------------------------------------------------- : 253
DrMTMR10      : -----------------------------IS-------DPQQQRKVEDRISSAIITKSHPLR-----------------------------------------------------------------------------------------------------------SDVFKSD--LDKN-------------------------------------------------------------------------- : 290
MTMR11_Hu     : ----------------------------FY-------TASDPNKEDIRAVELVLQAGHSD---------------------------------------------------------------------------------------------------VVLVD----------------------------------------------------------------------------------------- : 232
MTMR12_Hu     : ------------------------------------------KEQDQGILQIQKSFLDG-------------------------------------------------------------------------------------------------IYKTIHRPPYEIV---KTED----------------------------------------------------------------------- : 277
N001629628    : ----------------------------GRP-------ESIFLEKEETSLFRALSSIPANP-----------------------------------------------------------------------------------------------------EHKDVKVVDL---SKI------------------------------------------------------------------------ : 261
Tad54716      : --------------------------EDHG-------LRENCNEMEGRIFGIIKDPNKQKP----------------------------------------------------------------------------------------------FKTIELANRFPNYLATVWQR---------------------------------------------------------------------- : 330
M01745983C    : -------------------------QPLVGVRS--------KRSMEDEDMVAALRACAS----------------------------------------------------------------------------------------NSDQIAVFDARSMIAAG---GNQIMG-K-----------------------------------------GSENS--AFY------------------------ : 433
Tad51955      : -------------------------QPSVGPMG--------K----------------------------------------------------------------------------------------------------------------------------------G-K-----------------------------------------GTEDI--TNRY----------------------- : 460
Mbrevi5R3     : --------------------------PRVG-------FVGNRCAEDERYLDTLRGLVGSSDT----------------------------------------------------------------------------------------------LWIMDARPRRNAR---ANQLKG-M-----------------------------------------GFEST----------------------------- : 222
Tad62282      : ------------------------------------------------------------------------------------------------------------------------------------------------------------------------------------------------------------------------------------------------------------------------------------------- : -
Nv82921       : ------------------------------------------------------------------------------------------------------------------------------------------------------------------------------------------------------------------------------------------------------------------------------------------------------- : -
An18g03320    : -------------------------QPLVG-------VRQNRSIQDEALLAAIFSTSRSERPLASFTPPGFDSDSSNSTPENASRSHMLADLTVPDDLEAEMLTSV-----------------RG--DPEERPQIYGAQQHNLIVDARPTVNAF---AMQAVG-L-----------------------------------------GSENM--DNY------------------------ : 348
Fg05432.1     : -------------------------QPLSG-------ITRKTNVQDEKLVAASFNALLPPGSEDTTPLSSQADVSLVSSTTEPELSETERYEDELISKS-----------------SAVYDASGKRQVYGAQQSNLIVDARPTINAM---VNQVQG-M-----------------------------------------GSENM--DNY------------------------ : 332
Sc_YJR110W    : -------------------------QPLPG-------LINQRSIQDEKLVWESFNSFCNKDIRR-----------------------------------------------------------------------------------TKHVIVDARPRTNAL---AQMALG-G-----------------------------------------GTENM--DNYNFFLADNNMGVDKSL---------- : 316
SpNP593789    : -------------------------QPLVG-------IRQNRSAQDEKLVEAIFATSIIPGK-----------------------------------------------------------------------------------------------ENLIVDARPSTNAM---ANIAVG-A-----------------------------------------GTENM--DHY------------------------ : 266
Lb_143319     : -------------------------QPMVG-------ITQNRSVQDEKLIEAVFQSHWSPESRASN-----------------------------------------------------------------TPVYGATSTNLIIDARPTVNAV---ANSAKG-A-----------------------------------------GTENM--DHY------------------------ : 269
Um05646.1     : -------------------------QPMVG-------LKNARSIQDEKLIEAIFTSHHFADPESIMAKAAADASSSAASSASG--------------------------------------------APAVATVYGATTTNLIVDARPTVNAM---ANSAKG-A-----------------------------------------GTENM--EYY------------------------ : 322
ChXP666257    : -------------------------QPKSA-------FKRCIEDEIMITTISSFYKNNGDP--------------------------------------------------------------------------------------KEERPFAILDARPKLNAF---ANKATG-A-----------------------------------------GFENI--DHY------------------------ : 367
Pt16054001    : -------------------------QCRTG-------IGQNRSTEDECYLKYIQ-------YEEKE-----------------------------------------------------------------------------------------TRLRIYDARPHINAI---GQQVAG-K-----------------------------------------GYENT--LFY------------------------ : 288
Pt24407001    : -------------------------QCRTG-------IGQNRSTEDECYLKYMSQ-------YEEKE----------------------------------------------------------------------------------------IRLRIYDARPHINAI---GQQVAG-K-----------------------------------------GYENT--LFY------------------------ : 289
Pt26750001    : -------------------------QCRIG-------IGQIRSLEDELYLKQVSQQMVDEPENQQEE-------------------------------------------------------------------------------------IQLKIFDARSDVNSI---GQQTSG-K-----------------------------------------GFENP--MVY------------------------ : 297
T3692.m00019  : -------------------------QCRTG-------F-SSRSTEDEQMLRLIGD-PTNKDNRSSNN-----------------------------------------------------------------------------------VNVHIYDARPFMNAV---GNKLAG-K-----------------------------------------GYESN--SCY------------------------ : 294
T3707.m00240  : -------------------------QCKPG-------INSNRSIEDETFLKVIGC-LEKNSNIPK------------------------------------------------------------------------------------VVKIYDARPYLNAV---GQQITG-K-----------------------------------------GYEQK--QYY------------------------ : 305
Tp261179C     : -------------------------QPKVG-------LQGNRSAEDERYLYAIGDEAKRANLAADALAEGGVGGGTGAAGKPPMEFLRMLCGRNNESDLIL-----------------------------EGASSSCMLKIMDMRPKSAAM---ANRTQG-Y-----------------------------------------GYENT--NYYRG---------------------- : 400
At3g10550C    : -------------------------QPLVG-------LMMNMRSNLDEKLVAAFCSQLPGAKGERRK-----------------------------------------------------------------------------------------LYIADARPRKNAL---ANGAMG-G-----------------------------------------GSESS--SNYFQ----------------------- : 300
At5g04540     : -------------------------QPLVG-------LMMNMRSNSDEKLVASFCTQLAGHKGARRK-----------------------------------------------------------------------------------------LYIADARPRKNAL---ANGAKG-G-----------------------------------------GSESS--SNYLQ----------------------- : 300
O8g44260.1    : -------------------------QPLVG-------LMMNFRNNTDEKLVSALCTQIIDATGSLRK-----------------------------------------------------------------------------------------LYIVDARPRANAL---ANGAKG-G-----------------------------------------GSESA--SNYPR----------------------- : 296
O8g44260.2    : -------------------------QPLVG-------LMMNFRNNTDEKLVSALCTQIIDATGSLRK-----------------------------------------------------------------------------------------LYIVDARPRANAL---ANGAKG-G-----------------------------------------GSESA--SNYPR----------------------- : 296
Pop228298C    : -------------------------QPLVG-------LMMNMRSNTDEKLVAAICSQLGGDKGKRRK-----------------------------------------------------------------------------------------LYIADARPRANAL---ANGAKG-G-----------------------------------------GSESP--SHYFQ----------------------- : 268
Pop420099C    : -------------------------QPLVG-------LMMNMRSNTDEKLVAELCSQLGDEKKRRRK-----------------------------------------------------------------------------------------LYIADARPRKNAL---ANVAMG-G-----------------------------------------GSESS--SHYFQ----------------------- : 262
Sorghum       : -------------------------QPMVG-------LMMNFRNNADEKLVCALSSRTTDEKGSPRK-----------------------------------------------------------------------------------------LYIIDARPRANAL---ANGAKG-G-----------------------------------------GSESS--SNYPR----------------------- : 295
O35009001C    : -------------------------QPLVG-------LMMNMRSNTDEKIVAALCTQLAGARETRRK-----------------------------------------------------------------------------------------LYIADARPRKNAL---ANGAMG-G-----------------------------------------GSESS--SHYFQ----------------------- : 303
Phy12645      : -------------------------QPFTG-------FVFMLCSKEDEQLVAAVCNTA----NQRRK-----------------------------------------------------------------------------------------LYIADARPRKNAL---ANGVTG-G-----------------------------------------GSESS--ANYLQ----------------------- : 274
Phy71628      : -------------------------QPFT--------AFGMTRSTADEKLVAAVCDTI----NQRRK-----------------------------------------------------------------------------------------LYIADARPRKNAL---ANSAFG-G-----------------------------------------GSESS--ANYLQ----------------------- : 286
```

```
                    *       980         *      1000         *      1020         *      1040         *      1060         *      1080         *      1100         *      1120         *      1140         *      1160         *      1180         *      1200
SS          : -----------------------------------------------------------------------------------------------------------------------------------------------------------CCEEEEECCCCCH--------------------------------------------HHHHHHHHHHHHHHCCC----------- : 301
GL_8210     : -------------------QRHIADCALFASTQDN-----------------------------------FQYHRLGRVGGITEVLGV-------------------------------------------------------------------DPRNYILQDTEWMKVDEVQTTRSQQDFVADNQPPAELVSRDRIREDGVDHESSECDISGVEQLSPSIDFNTIAVPQYVGN----- : 414
GL_13875    : -------------------------------------------------------------------------------------------------------------------------------------------------------------SRTIYLNIQNI--------------------------------------------HVVRDSYEAMRKAALEFILTREANFR-- : 374
LmF20.1480  : --------------------------------------------ESTPVNGSGCTSVRTPPSTVSKAARKFTVTAAGDAANVATTPLMETSHGAVMGASTNANAAKVLLVADCRPQITAAGNA--------------------QLGGGYESGSYYTFCQTNFFEIDNI--------------------------------------------FGVAKSFEKLRSMLA------------- : 517
Tb.1.3300   : --------------------------------------------------------------------------------------------------EDLPRRQRTLVVMDCRPYSAALGNA--------------------NLGGGYENGSTHYFCDVRFGNIENI--------------------------------------------HAVSKSFAKLKELIQ------------- : 385
GL_112811   : ------------------TKLPFLEELAMA----------------------------------------------------------------------------------------------------------------------FNTMWSLNLVQPTQFFSSEL---------------------------------------PANWNLQQKGDTDLYSVAADLFDTFHIKTIKED : 384
LmF12.0320  : QLRGDESGRLETQYVQNAGEPPYAAVVAAAELSATAKRPASGLGVASLLPRLQGCSPSTPQSARDLSSGADAEAAIKLSAGESGDSDGNGESGGPRDGAAAEDYAANTTAATSAPSRPPFSLGGVRWSAIFSVMAPVGSASVTAMMPAAQHEWLAYIAMLLNTAVVAA-----------------------------------------RRVCGVPTDPLYVPALQHNGPLANCFSYIVG : 582
Tb.6.870    : -------SLCEEIFTHNPNFPTAVMKCATAPKRQNT--------------------------------------------QAGGPTSSLAQLRAQ----------------------------------------------ENQPLGKAKMAWVEHIRGLLRTAEDAA-----------------------------------------RLVAGVSEENALFPAKTDVGTSSTMYMWMRA : 383
Dd0186027   : -----------------------------------------------------------------------------------------------------------------------------------------------------------YNCDIEFLGIANI--------------------------------------------HSMRDSINKLEQFCQSNN---------- : 332
Dd0190143   : ----------------------------------------------------------------------------------------------------------------------------------------------------------YPNCKLVFMNIPNI--------------------------------------------HVIRKSLEKLTSSMRSSTRDKD------ : 346
Dd0190271   : ----------------------------------------------------------------------------------------------------------------------------------------------------------YPNCKLIFMNIPNI--------------------------------------------HVIRKSLEKLVLAMKSSARDKD------ : 341
Dd0204242   : ----------------------------------------------------------------------------------------------------------------------------------------------------------YENCIIEFLGIENI--------------------------------------------HTMKESFKKLREAVFGNYNNIINNVNIN : 325
Dd0216866   : ---------------------------------------------------------------------------------------------------------------------------------------------------------LYQNVELRFLGIGNI--------------------------------------------HVMRDSYRKLYNLVQNAE---------- : 345
Dd0231579   : ----------------------------------------------------------------------------------------------------------------------------------------------------------YNNCILKFLNIENI--------------------------------------------HKMRHSQKKLFKSIMSDSVIYGENNNNN : 316
Dd0238869   : ----------------------------------------------------------------------------------------------------------------------------------------------------------YSCCNIDFLNIGNI--------------------------------------------HVMRDSYEKLKSTCIDSLGGIKEIKNSN : 423
Dd0238873   : -----------------------------------------------------------------------------------------------------------------------------------------------------------PGCTLEFLNIANI--------------------------------------------HSMRHSLWKIKDAAVSSID--------- : 326
Dd0266510   : ---------------------------------------------------------------------------------------------------------------------------------------------------------HYSQCQIESECLSNI--------------------------------------------HELRESQLKLFKVIRNWN---------- : 395
EHI_016430  : -----------------------------------------------------------------------------------------------------------------------------------------------------------KNSEIIFLEIDNI--------------------------------------------HKMRESFLELTSVTKKLSYCNVGAT--- : 365
EHI_024380  : -----------------------------------------------------------------------------------------------------------------------------------------------------------KKTQLKFLNIPNI--------------------------------------------HTVVDCFIKMRNALFIRDTL-------- : 371
EHI_031310  : -----------------------------------------------------------------------------------------------------------------------------------------------------------GNATITFLNIDNI--------------------------------------------HKMRDAEDGLFTIIE------------- : 343
EHI_049780  : -----------------------------------------------------------------------------------------------------------------------------------------------------------TFCSIEFLNIPNI--------------------------------------------HRVKEAFNECYDIFLTQDETM------- : 328
EHI_070120  : -----------------------------------------------------------------------------------------------------------------------------------------------------------PFATIEFESIDNI--------------------------------------------HVVREGWQKLCWYCMQLPEHYKTS---- : 377
EHI_087780  : -----------------------------------------------------------------------------------------------------------------------------------------------------------KNCIIDHLNLPNI--------------------------------------------HVVFSAYQKMINGIINRSKK-------- : 370
EHI_104710  : -----------------------------------------------------------------------------------------------------------------------------------------------------------PFCEFQYLSIENI--------------------------------------------HEVRNAYYKMLKAILMMPHNSY------ : 356
EHI_161190  : -----------------------------------------------------------------------------------------------------------------------------------------------------------PFVKVEFENIDNI--------------------------------------------HVVREVWTNVALAAFSWEGELEEQ---- : 355
Dd0191503   : -----------------------------------------------------------------------------------------------------------------------------------------------------------IGCQIEFLNLPPP--------------------------------------------NKVRERFNRLLHLHLGNP---------- : 324
Dd0191512   : ----------------------------------------------------------------------------------------------------------------------------------------------------------DVKLYNLFVNKEEQ--------------------------------------------LQSSTSSSPSTSLNNSSNNLNKEFQQIH : 498
EHI_048230  : -----------------------------------------------------------------------------------------------------------------------------------------------------------TFPDIALKDNETY--------------------------------------------KTMKDLEMRLFDSYTSNANE-------- : 300
EHI_078170  : -----------------------------------------------------------------------------------------------------------------------------------------------------------PQEKNDSMILKTS--------------------------------------------DEVNIIWNKIVDSAKKFTETT------- : 320
EHI_107230  : -----------------------------------------------------------------------------------------------------------------------------------------------------------EKIKCVEVNISDL--------------------------------------------ETFDKTFNDILNNLSNNKDSL------- : 291
EHI_135010  : -----------------------------------------------------------------------------------------------------------------------------------------------------------STYNTNPSLLKTC--------------------------------------------DTLQDSFHNKVVKLMNDVNEK------- : 337
EHI_137960  : -----------------------------------------------------------------------------------------------------------------------------------------------------------YQCTISKYDIIGE--------------------------------------------HEISEMVNKIQELLPTVNTKNNQ----- : 230
EHI_141820  : -----------------------------------------------------------------------------------------------------------------------------------------------------------EIIDLSYLDDKIL--------------------------------------------EEEMNNYYRFITKIKKE----------- : 284
EHI_151670  : -----------------------------------------------------------------------------------------------------------------------------------------------------------KALKIYCKSIDNT--------------------------------------------DNIDNIFQDLKNFVCEESINSE------ : 287
EHI_185230  : -----------------------------------------------------------------------------------------------------------------------------------------------------------IKCELKPKNV-------------------------------------------------IEKCFKKIQDIVPNVNFDLNK----- : 290
EHI_197200  : -----------------------------------------------------------------------------------------------------------------------------------------------------------KMTQFAYQTLEPA--------------------------------------------KEIAKKLLTQ------------------ : 251
EHI_140980  : -----------------------------------------------------------------------------------------------------------------------------------------------------------PTMKIREIPCFDR--------------------------------------------KTLMKYYHQFIEYINQPE---------- : 295
EHI_188050  : -----------------------------------------------------------------------------------------------------------------------------------------------------------PFCTFENLALDSI--------------------------------------------KDIKQAFYKMIKTIVFERRDVY------ : 356
MTM1_Hu     : -----------------------------------------------------------------------------------------------------------------------------------------------------------HNAELFFLDIHNI--------------------------------------------HVMRESLKKVKDIVYPN----------- : 300
MTMR1_Hu    : -----------------------------------------------------------------------------------------------------------------------------------------------------------PNAELVFLEIHNI--------------------------------------------HVMRESLRKLKEIVYPS----------- : 300
MTMR2_Hu    : -----------------------------------------------------------------------------------------------------------------------------------------------------------QNAELVFLDIHNI--------------------------------------------HVMRESLRKLKEIVYPN----------- : 301
DrMTMR2     : -----------------------------------------------------------------------------------------------------------------------------------------------------------QNAELVFLDIHNI--------------------------------------------HVMRESLRKLKEVVYPN----------- : 300
N001636543  : -----------------------------------------------------------------------------------------------------------------------------------------------------------QNTEVIFLDIANI--------------------------------------------HVMRESLRKLKDICFPN----------- : 300
Tad38469    : --------------------------------------------------------------------------------------------------------------------------------------------------------EKYTNMELEFLDIENI--------------------------------------------HVMRDSMNKLRDVCYAID---------- : 298
MTMR3_Hu    : -----------------------------------------------------------------------------------------------------------------------------------------------------------PNCEVVFMGMANI--------------------------------------------HSIRRSFQSLRLLCTQMP---------- : 340
MTMR4_Hu    : -----------------------------------------------------------------------------------------------------------------------------------------------------------PNCEVVFMGMANI--------------------------------------------HAIRNSFQYLRAVCSQMP---------- : 334
N001631983  : -----------------------------------------------------------------------------------------------------------------------------------------------------------PNCEVMFMNLANI--------------------------------------------HGIRRSFHSLRALCSGSY---------- : 353
Tad64213    : -----------------------------------------------------------------------------------------------------------------------------------------------------------PLVSSGSYSVEASS-------------------------------------------SQRRNSFQIIDARSYAAALANRAMGGGL : 339
MTMR5_Hu    : -----------------------------------------------------------------------------------------------------------------------------------------------------------QQWELVPIEVFEA--------------------------------------------RQVKASFKKLLKACVPGCP-AA------ : 494
DmMTMR5/13  : -----------------------------------------------------------------------------------------------------------------------------------------------------------FCADFIPVDYPDI--------------------------------------------RQSRPAFKKLIRACMPSHN-TN------ : 486
DrMTMR5     : -----------------------------------------------------------------------------------------------------------------------------------------------------------QQWEVVPIEVFDV--------------------------------------------RQVKNSFKKLMKACVPSSLS-------- : 488
MTMR13_Hu   : -----------------------------------------------------------------------------------------------------------------------------------------------------------LNCEFVPVEFHEI--------------------------------------------RQVKASFKKLMRACIPSTI-PT------ : 495
Nv109357    : LYVLG-----------------------------------------------------------------------------------------------------------------------------------------DKAHLRNVKQEAYPKCEFVPVELPDVR-------------------------------------------AVRACFKKFLRACCPATDS-------- : 505
Tad51481    : ---------------------------------------------------------------------------------------------------------------------------------------------------AIKQVKNEQNSKCEFIPVEYPES------------------------------------------RSVRSSFKKLLRCCCPSNAE-------- : 553
M001750622  : -----------------------------------------------------------------------------------------------------------------------------------------------------------TFIDVTMASRPLI--------------------------------------------TSVATIHEMARELSVLAAG--------- : 393
MTMR6_Hu    : -----------------------------------------------------------------------------------------------------------------------------------------------------------SNIRFQFVGIENI--------------------------------------------HVMRSSLQKLLEVNGTKG---------- : 292
MTMR7_Hu    : -----------------------------------------------------------------------------------------------------------------------------------------------------------SNIKFQFIGIENI--------------------------------------------HVMRNSLQKMLEVCELKS---------- : 294
DrMTMR7     : -----------------------------------------------------------------------------------------------------------------------------------------------------------TNIKLQFIGIENI--------------------------------------------HVMRNSQQKLIDVGDMAAP--------- : 294
MTMR8_Hu    : -----------------------------------------------------------------------------------------------------------------------------------------------------------ANIRFRFMGIENI--------------------------------------------HVMRSSLQKLLEVCELKT---------- : 294
N001626810  : -----------------------------------------------------------------------------------------------------------------------------------------------------------ENVKFQFVGIENI--------------------------------------------HVMRQSLFKMLEVVGESPK--------- : 296
Tad56124    : -----------------------------------------------------------------------------------------------------------------------------------------------------------PNVLYRFRGIQNI--------------------------------------------HVMRESLQKLSEVIENPALN-------- : 294
M01746603C  : -----------------------------------------------------------------------------------------------------------------------------------------------------------TNCRLEFHGIENI--------------------------------------------HTMRGSL--------------------- : 278
MTMR9_Hu    : -----------------------------------------------------------------------------------------------------------------------------------------------------------PQWRRIHKSIERY--------------------------------------------HILQESLIKLVEACNDQT---------- : 289
DrMTMR9     : -----------------------------------------------------------------------------------------------------------------------------------------------------------PQWRRIHKAIERS--------------------------------------------NILQESLIKLVEACNDQS---------- : 289
N001625874  : -----------------------------------------------------------------------------------------------------------------------------------------------------------PQWKRSNHNIEKH--------------------------------------------TVLIDSVCKLMEACFDQN---------- : 291
Tad28926    : --------------------------------------------------------------------------------------------------------------------------------------------------------CYYPQWHRIHRGIENY--------------------------------------------NSLKDSLTKLVEATSDFDCS-------- : 292
M01749550C  : -----------------------------------------------------------------------------------------------------------------------------------------------------------YGWSRKMLDIAAW--------------------------------------------PILADSLSKLLEACQDPS---------- : 122
MTMR10_Hu   : --------------------------------------------------------------------------------------------------------------------------------------------------------------------LPNI--------------------------------------------QEVQAAFVKLKQLCVNEP---F------ : 276
DrMTMR10    : --------------------------------------------------------------------------------------------------------------------------------------------------------------------LPNI--------------------------------------------QDIQAALLKLRQICVIESFE-------- : 314
MTMR11_Hu   : ----------------------------------------------------------------------------------------------------------------------------------------------------------------TMDELPSL--------------------------------------------ADVQLAHLRLRALCLPDSS--------- : 259
MTMR12_Hu   : ----------------------------------------------------------------------------------------------------------------------------------------------------------------LSSNFLSL--------------------------------------------QEIQTAYSKFKQLFLIDNSTEF------ : 307
N001629628  : --------------------------------------------------------------------------------------------------------------------------------------------------------------------CPSI--------------------------------------------QQLSQSYLRVRELCVLDTTKNF------ : 287
Tad54716    : -----------------------------------------------------------------------------------------------------------------------------------------------------------IYTDNGIVRVGNVIQP-----------------------------------------KELSSGYNNLKYHCLKGSDRKYWN---- : 370
M01745983C  : -----------------------------------------------------------------------------------------------------------------------------------------------------------VGAQVFFMEIGNI--------------------------------------------HTMRASHDKLLELCIG------------ : 462
Tad51955    : -----------------------------------------------------------------------------------------------------------------------------------------------------------IGCRLIYLNIANI--------------------------------------------HAMRESFDKLLAICES------------ : 489
Mbrevi5R3   : --------------------------------------------------------------------------------------------------------------------------------------------------------AHYQNCIMTFLNIQNI--------------------------------------------HVMRDSLKKMLELCWPIP---------- : 256
Tad62282    : ------------------------------------------------------------------------------------------------------------------------------------------------------------------------------------------------------------------------------------------------ :   -
Nv82921     : ----------------------------------------------------------------------------------------------------------------------------------------------------------YQGCSLVFLDIPNI--------------------------------------------HAVRDSLDRLQAVCESSSQKK------- :  35
An18g03320  : -----------------------------------------------------------------------------------------------------------------------------------------------------------KFATKAYLGIDNI--------------------------------------------HVMRDSLSKVIDALKDSD---------- : 379
Fg05432.1   : -----------------------------------------------------------------------------------------------------------------------------------------------------------KFARKIFLSIENI--------------------------------------------HVMRSSLNKVVDALKDADIS-------- : 365
Sc_YJR110W  : ---------------------------------------------------------------------------------------------------------------------------------------------------------KLPTVTRLFLGIDNI--------------------------------------------HIVSNTAAYMTEVICQGGDLN------- : 352
SpNP593789  : -----------------------------------------------------------------------------------------------------------------------------------------------------------RFAKKIYLGIDNI--------------------------------------------HVMRDSLNKIVNALKNTDIS-------- : 299
Lb_143319   : -----------------------------------------------------------------------------------------------------------------------------------------------------------KDSKKVYLGIDHI--------------------------------------------HTMRESLAKVVEALRDADSLLAT----- : 305
Um05646.1   : -----------------------------------------------------------------------------------------------------------------------------------------------------------KGCKKSYLGIENI--------------------------------------------HVMRDSLNKLTDALRESEPIATFGMRVE : 363
ChXP666257  : -----------------------------------------------------------------------------------------------------------------------------------------------------------TNCKLEFLNIENI--------------------------------------------HKVRDSWNKLVSVIQKTSSNQDLNVGNA : 408
Pt16054001  : -----------------------------------------------------------------------------------------------------------------------------------------------------------RRCSIDFLDIHNI--------------------------------------------HKVRDSQTKLLKA--------------- : 314
Pt24407001  : -----------------------------------------------------------------------------------------------------------------------------------------------------------RRCSIDFLDIHNI--------------------------------------------HKVRDSQTKFISI--------------- : 315
Pt26750001  : -----------------------------------------------------------------------------------------------------------------------------------------------------------KNCSIEFLEIQNI--------------------------------------------HKMRESQTKLLKN--------------- : 323
T3692.m00019 : -----------------------------------------------------------------------------------------------------------------------------------------------------------KNCDIQFLDIHNI--------------------------------------------HKVRDSYNKLVSV--------------- : 320
T3707.m00240 : -----------------------------------------------------------------------------------------------------------------------------------------------------------RNGDIEFLDIHNI--------------------------------------------HKVRESYKKMAAA--------------- : 331
Tp261179C   : -------------------------------------------------------------------------------------------------------------------------------------------------------------TTINFYGIGNI--------------------------------------------HAVRDAYQKICALC-------------- : 425
At3g10550C  : ----------------------------------------------------------------------------------------------------------------------------------------------------------SEI---VFFGIDNI--------------------------------------------HAMRESFSRVRDYLDMHGTTSSDGRSSF : 339
At5g04540   : ----------------------------------------------------------------------------------------------------------------------------------------------------------SEI---VFLGIDNI--------------------------------------------HAMRESFSRLRDYLDMHGTTSSDGTSSF : 339
O8g44260.1  : ----------------------------------------------------------------------------------------------------------------------------------------------------------SEV---LFLGIQNI--------------------------------------------HTMRDSLFRLRDYVDTHGSVSSNGTSSA : 335
O8g44260.2  : ----------------------------------------------------------------------------------------------------------------------------------------------------------SEV---LFLGIQNI--------------------------------------------HTMRDSLFRLRDYVDTHGSVSSNGTSSA : 335
Pop228298C  : ----------------------------------------------------------------------------------------------------------------------------------------------------------SEV---VFFGIDNI--------------------------------------------HAMRESLSRLRDYLDTHGSTSSDGTLSL : 307
Pop420099C  : ----------------------------------------------------------------------------------------------------------------------------------------------------------SEV---VFFGIDNI--------------------------------------------HSMRESLSRLRDYLDAHGTTSSDGTLSL : 301
Sorghum     : ----------------------------------------------------------------------------------------------------------------------------------------------------------SEV---LFLGIQNI--------------------------------------------HTMRDSLSRLRDYVDAHGSISSNGSPSA : 334
035009001C  : ----------------------------------------------------------------------------------------------------------------------------------------------------------SEI---VFFGIDNI--------------------------------------------HAMRESFARLRDYLDTYGTASSDGMSSF : 342
Phy12645    : ----------------------------------------------------------------------------------------------------------------------------------------------------------SEARSVIFLGIDNI--------------------------------------------HGMRDSLTKMRDFLDIHGAASSDGSSSL : 316
Phy71628    : ----------------------------------------------------------------------------------------------------------------------------------------------------------SEV---IFLGIDNI--------------------------------------------HGMRDSLTKMRDFLDTHGASSSDGSSSL : 325
```

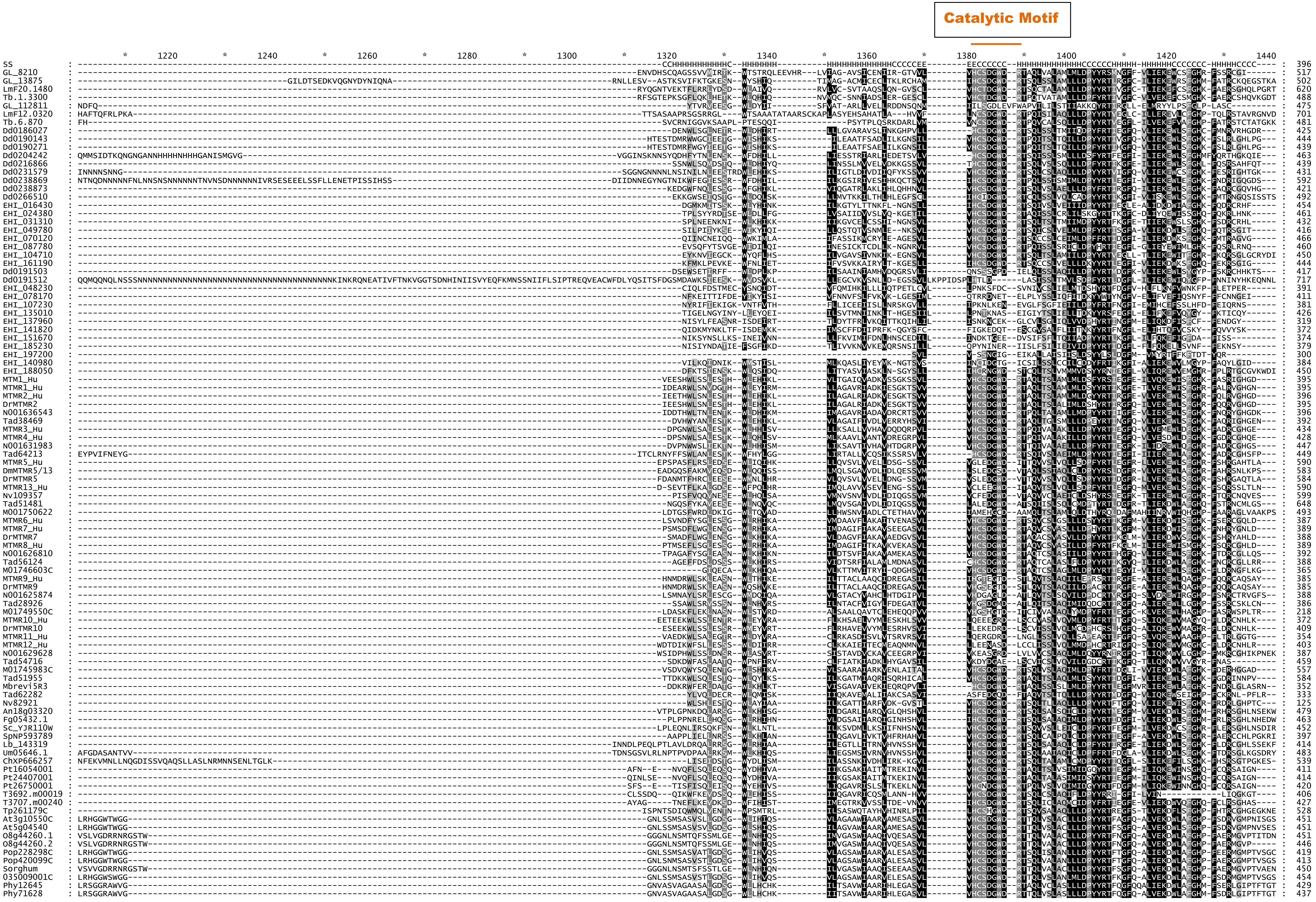
Catalytic Motif

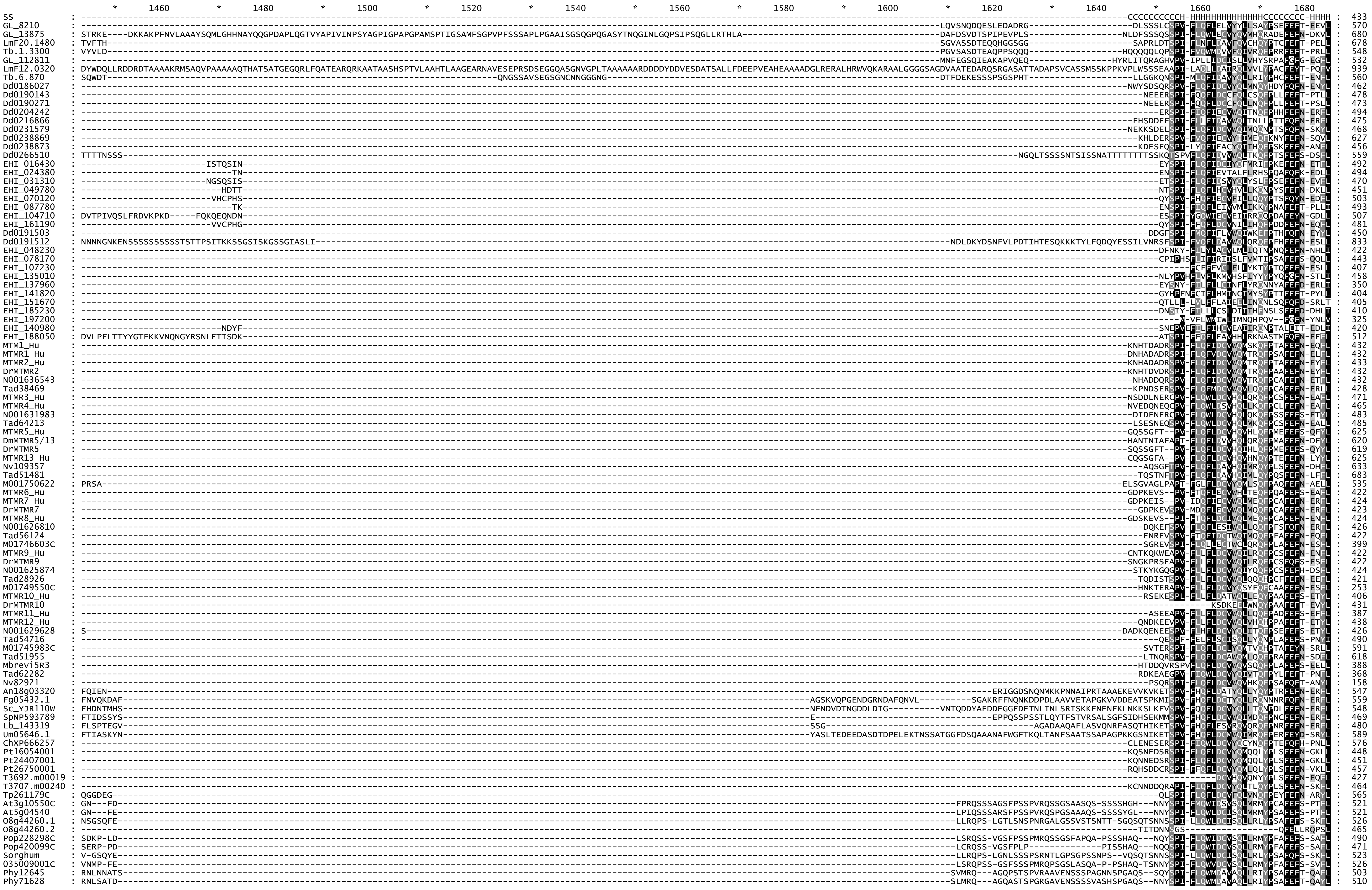

```
                   *      1700         *       1720         *       1740         *       1760         *       1780         *       1800         *       1820         *       1840         *       1860         *       1880         *       1900         *       1920
SS            : HHHH-HHHHHCCCCCCCCCCHHHHHHH----CCHHHHHCCHHHHHHC----CCCCCCCCCCCCCCCCCC---- :  491
GL_8210       : KYLA-YHTYSARFGTFIGDCELDRLLCQ----LPIRTAS--IWDHLLAC----KDHYTNKGYSPESILK----KNGWV---- :  633
GL_13875      : LYIA-HHAYSGWHGTFIFDNERDRTQHL----LSTITTS--VWDEFITN----SAYYLNPHYKIPAPLN----VMKTRTGLE---- :  747
LmF20.1480    : AHLS-EVVYSCLYGTFLCNGEQERHLEG----VRLRTAS--VWTDVLRRTQREKAGKI----PLCFVNIHYDAVTAWR----FISQRRRGRVE---- :  756
Tb.1.3300     : EYLS-TEVYACLHGTFLCNGEKERMFEG----VRLNTAS--IWTDIVRTALREREGKA----PLYFVNPAYDSAAAWE----FISKRKGCGIN---- :  626
GL_112811     : MHLS-AIIYSNLYGDFLFDSEAERYF----LAIPELTDSLYSQEA----IELLSKRFETPIVVD---- :  587
LmF12.0320    : CLLL-DLFHSQLISTFSVNCEQQVEAQR----VAEGTLSLSQWCALLLSTTTATPS----AAYAAAPVTVQPSPLRSTLDTHSDGGGDANTVLLLSSASHSRAPATPGSLRQQLPRSTAAALSASHADPPSEVGGAEASVGAAVSVDASFHRLDSAFLMGDAGLEQEESGENAVDSCRRSCSDAV : 1115
Tb.6.870      : LLIV-DILNAGIVGTFAVNCEADIKRWG----VEKHTLSLSQLIALVLATTIPTEGE----QLTSLESSIDVRRAYD----DGEYRVTNASVDTE---- :  642
Dd0186027     : ITIL-DALYSCQFGTFLCNSEKERQT----IKKDTVSLWSYINSNP----LTPYLNPFYISYEHETSR---- :  521
Dd0190143     : LFIS-DNIHSCKYGTFLNNCEKDRVQ----DQVKFKTTSIWSEVHFN----IHKFLNPYYNPQDIQS---- :  536
Dd0190271     : LFIS-DNIHSCKYGTFLNNCEKDRIQN----QVKTKTISIWTEVNLN----VNKFLNPYFNPQDIQS---- :  531
Dd0204242     : IKIL-DALYSCQYGTFLYDCEKERSK----AFNTPSFWAYAETN----RQLFINPLYVLPKQQQQQQQQTSSSSSHSSSNNVNGSSSSGSSSKSS---- :  581
Dd0216866     : IKIL-DSVYNCRYKQFLFNNEKDRLS----YQKNYPNGLVSLWSVVNSNLEDHVNSFY----IADPKPIYENFFMGDIQ---- :  545
Dd0231579     : LEII-HHLYSCRFGTFLFDCQEKRVS-A----NLMSKTTSIWSYINQN----HSPYFNKNYIYNSNEP---- :  526
Dd0238869     : VEILVNGLYSNRFGTFLYNTVRERIN----KVQLETASIWSLILSN----YSFYINQNYTRMDKV---- :  685
Dd0238873     : KTIL-YHSYSGKYGNFLFNTEKERVQN----HIYGKTISLWAEMDGN----LKEYINPLYQPTGIKERD---- :  516
Dd0266510     : SVIL-HHLNSNLFGTFLYDSEKERQQ----NNLPTETQSLWTLLLSAQ----KNSSLLNPLFNQQLSNETSSTTN---- :  625
EHI_016430    : IEII-QQTYSCYFCNFLFNC-----V----EPINPRPESLWNILN-NK----EIQCRFVNPSY----HPSTSI---- :  546
EHI_024380    : VKIA-EEATCPTTITFVRNCEKERSA----LS-CDNYLEFFTQEL----KHYPTSFDSN-------KLEP---- :  547
EHI_031310    : KELL-FQLYSCYFCNFRFNCWNE--F----NSFGNRVMNFYSFLDYEE----EILFSFSNELF----NPQLKV---- :  528
EHI_049780    : QFLA-DELFTFKYGTFAFNCEKDRKE----IKASLYSASIWDDVI----NMTEFINEHF----KLSSNP---- :  507
EHI_070120    : IEID-DAVFCHNYGTFLFDTDCEREK----YHVNERTPSLWSMII----ENRELFTNHQY-----ILSD---- :  558
EHI_087780    : KDLA-VESVCPTTGTFLYNCEKERRK----YDVCTTRMSFFQHII----QNKTVYINPNAT---LKKYNE---- :  551
EHI_104710    : IFLL-DSLYNCQFANYL---EYPNTC----SCNISNLLSMVPYVY----SHRIKFTNTSF----KAVADD---- :  561
EHI_161190    : LELN-DAIFCSDFGTFMFNCEKTRKE----SGVELQTPSFWEYVV----GQKCKFINPSY----KDETEE---- :  538
Dd0191503     : LTLL-DNVYNSRFGTFLCNNYKERMEN----NVYSSTKSFWSFQQQN----QSRFTNLFYRPSPSSS---- :  508
Dd0191512     : LLLV-KESFSGRFCNFLYSEALRDSE-R----KFLKRSPSIWTFINQN----KSTFINLLYKHSANSSSSSSSTNKLPSPISPRQSFIFKQEDFNE---- :  919
EHI_048230    : KFFY-YHCYSKRFIEFL----PFQVYGLASVYSYLQ----IHKNEFINTNY------LERQ---- :  468
EHI_078170    : NYLE-LICMSGRLR----FNESRSQRSIVRLLVMKN----KLKNQRYNSSTN---VSLIDM---- :  492
EHI_107230    : HFIF-YHYNSRVFKDFN----QNDMSSLQSYIQ----SHLNDFKNEFY----ESTLEV---- :  452
EHI_135010    : HFLY-QHCNSGRFSAF----EGSSTQGVFVYLY----SNKEFFVNNSFVNEECDFYHK---- :  507
EHI_137960    : QLTF-HSIFSHRFYEFN----HSQVNSKSSFISLVQ----YYSSEFVNPLY----QRSNEL---- :  398
EHI_141820    : SFLT-YHTFSHRFSTI----SSQKNPTQSLYSYLL----NHKIQFIKSDFDNFAFDIMEY---- :  455
EHI_151670    : YFWI-THIGSRRFNNNNE----NGKCDQLSAIINYIY----SDSSGFINPFY----KPIQTE---- :  454
EHI_185230    : RFLF-CSYLSNRFSEFN----EKIIDSRNSIIRYIL----VYKSYFFNISY----KPNELS---- :  458
EHI_197200    : KTLF-YHITALRFNDDL---- :  341
EHI_140980    : IFIL-DELYKGKCNTFLFNSEKERRR----ECVDGKTLSLYDLII-QE----LKLSKFKNYNF-----DVTSS---- :  478
EHI_188050    : IFIA-DSLYDGRFTTFFENTI----K----TEDINPRNNLYEYVL----EHMTDFINSNY-----KESSM---- :  564
MTM1_Hu       : IIIL-DHLYSCRFGTFLFNCESARER----QKVTERTVSLWSLIN----SNKEKFKNPFYTKEINRV---- :  490
MTMR1_Hu      : ITIL-DHLYSCLFGTFLCNCEQQRFK----EDVYTKTISLWSYIN----SQLDEFSNPFFVNYENHV---- :  490
MTMR2_Hu      : ITIL-DHLYSCLFGTFLCNSEQQRGK----ENLPKRTVSLWSYIN----SQLEDFTNPLYGSYSNHV---- :  491
DrMTMR2       : ITIL-DHLYSCLFGTFLCNSEQQRLK----EEIPKRTVSLWSFVN----SQLEEFVNPLYVHYSSHV---- :  490
N001636543    : ITIL-DHLYSCMFGTFLYNSESQREK----EDVKTRTTSLWSYIN----TSMMEFKNPLYAPYLHQHV---- :  491
Tad38469      : IAIL-DHLYSCLFGTFLYDCERVREEHN----LKTKTFSLWDYINLN----VEDYKNVQYTRHEHVIY---- :  487
MTMR3_Hu      : VKLV-QHTYSCLFGTFLCNNAKERGE----KHTQERTCSVWSLLR----AGNKAFKNLLYSSQSEAV---- :  529
MTMR4_Hu      : VKLV-QHTYSCLYGTFLANNPCEREK----RNIYKRTCSVWALLR----AGNKNFHNFLYTPSSDMV---- :  523
N001631983    : IKLL-HHTYSCLFGTFLGNNEHGRHT----DAIKARTLSVWSYILP----RKKDFRNLLYAGGGDQV---- :  541
Tad64213      : IKLA-YHSYSCLFGTFLFNNVHERIQ----HKVAQRTKCFWRFVRTQ----REKFCNFLYDLYDKQVLN---- :  545
MTMR5_Hu      : KFLG-YHHVSRRFRTFLLDSDYERIELGLLYEE----KGERRGQVPCRSVWEYVDRLS----KRTPVFHNYMYAPEDAEV---- :  696
DmMTMR5/13    : RFLA-YHSVSCRFRTFLFDCELERSDSGIAAMEDKRGSLNAKHMFGAGGMATNGSDDECSVYPLDIRSQRAPAPLNRIGHSIFDYIERQH----NKTPIFYNFLYSGDKSVT---- :  727
DrMTMR5       : KFLA-YHYVSNRFRTFLLDSDYERIELGVLYEE----KGERKSTQVCKSVWDYIDRLN----KKTPIFYNYMFSPEEEEV---- :  690
MTMR13_Hu     : KFLA-FHYVSNRFKTFLLDSDYERLEHGTLFDD----KGEKHAKKGV-CIWECIDRMH----KRSPIFFNYLYSPLEIEA---- :  695
Nv109357      : CTLA-YHHVSMRFRTFLMDSEHERYQAG----WMIEEEGKRRGKSLWDYIDAQN----QRSPAFYNFMYSPDNQKVLR---- :  702
Tad51481      : ETIA-YHCCSSRFVTFMFDCERDRHYLEKSVLANESE----SFEKCLSPLKNTIWNYIDDVHR----NSTQFYNFNYSVHKMTQILR---- :  761
M001750622    : EAIF-HHSYSARFCNFLCNDQAYRDR----LGIDMDDLFAFVMDAH----AKTPQYANLMYSACNAHVLRTNAALPMMQL---- :  606
MTMR6_Hu      : LQIH-EHIHSCQFCNFLGNCQKEREE----LKLKEKTYSLWPFLL----EDQKKYLNPLYSSESHRF-TV---- :  482
MTMR7_Hu      : IHIQ-HHIYSCQFCNFLCNSQKERRE----LKIQERTHSLWAHLW----KNRADYLNPLFRADHSQTQGT---- :  485
DrMTMR7       : IQLH-MLIYSCQYCNFIGNCQKERRD----LKLQERTHSLWPHLW----ENRAEYTNPLYRTDHSQTQGV---- :  484
MTMR8_Hu      : LEIH-DHVFSCQFCNFLGNCQKDRED----LRVYEKTHSVWPFLV----QRKPDFRNPLYKG-FTMY-GV---- :  483
N001626810    : LTLH-DHVYSCQFGNFLGNCQKERRD----LGLPKRTYSLWGYMWQN----ISDYANPLYREVTQDDA---- :  485
Tad56124      : LTIH-DHVYSCQFCNFLGNCQQDRRK----ESLSEKTYSLWGYVCKQ----LATYLNPYYDPNFNADKRG---- :  483
M01746603C    : IAVH-DCLHSCKFGTFLGNCEQARRD----AKLHERTQSAWSYLYS----RKSEFINPFYDASQDPEGGV---- :  460
MTMR9_Hu      : IMLF-EHAYASQFGTFLGNNESERCK----LKLQQKTMSLWSWVNQP----SELSKFTNPLFEANNLVI---- :  482
DrMTMR9       : IMLF-EHTYASQFGTFLGNSVAERAK----LQLSHKTVSLWSWVNRP----QELERYLNPLYEANNLVI---- :  482
N001625874    : ICLF-EHAYYSQFGTFLCNNEKNRVT----VKLKEKTISLWSYLNQD----EILEDMLNPMYERNESVL---- :  484
Tad28926      : RVLG-HKAYSSEFGTFLYDSHAQRLQ----AQLPKRTCSLWSYINQAT----VINDYINPSYEMRKQIIRPS---- :  484
M01749550C    : MELY-MQSYSCVFGTFVGDCEAMRAA----EQLDKKTRCFWRHAPQP----EQRPRFANPLYDAAVNKGT---- :  314
MTMR10_Hu     : AVLY-DSTRISLFGTFLFNSPHQRVK----QSTEFAISKNIQLGDEKGLKF----PSVWDWSLQFTAKDRTLF---- :  470
DrMTMR10      : TVLG-DSMWVPVFSTFLFNCPRQRAEHSR----DFASSKSIHLGQDQALRF----PPVWDWSQQFSLKDQTLFNNPLYVGKIATCLQNGTVKTFTHRRSKKNYSSTVRGLPSLMRNGSLGPNDTLTRRNSLVLRLRSDLSQVREQPETPSERFVRDFFSRAVDLQGL---- :  589
MTMR11_Hu     : LALH-DSVRVPDTLTFLRNTPWERGKQSGQLNS----YTQVYTPGYSQPPAGNSFNLQ----LSVWDWDLRYSNAQILQF---- :  458
MTMR12_Hu     : TVLS-DSLYIPIFSTFFFNSPHQKDT----NMGREGQDTQSKPLNL----LTVWDWSVQFEPKAQTLLKNPLYVEKPKLDKGQRKGMRFKHQRQLSLPLTQSKSSPKRGFFREETDHLIKNLLGKRISKLINSSDELQDNFREFYDSWHSKSTDYHGL---- :  587
N001629628    : LGML-DSMHACLFDEFLFDCEHQRQKYIN----SEFGGAPMASLWDFLAEQLS----DSQNTAAFINPLNEFKRLSS---- :  494
Tad54716      : SHLL-DISLCGLTETFSYNNEYERCRYNYVRKG----EGNVSKRTGIVLPSAWNLCYLN----WKEVFNERYIMNQVLQRIYG---- :  564
M01745983C    : VFLA-DYSRAGWFCNFQCNSHRERRAR----RLSQTTTSIWSAVEADR----PRFTSSQYAPGGSDV---- :  649
Tad51955      : LRIA-DYFGSGWFCNFMFNCERERKEA----NLQENTPSLWAHIIADQ----ENFLNVKYLGTTEEPVVNDDEEDISNS---- :  688
Mbrevi5R3     : LDLS-DSMFTCYFGTFLFNSERERRQ----LPEKTFSVWDQVLQDR----VRYTNILYSP---- :  439
Tad62282      : HDIA-YHVYSCRFGTFLSNDVFDDEK----TLITEY-KELCNTLDIK----FDKKLYIDAHLNNNERC---- :  426
Nv82921       : LKLA-DHVNSQWFCNFLYNNVQERHH----AFITRTTVSLWSHLN----AVKDNYTNSIYQPTETLV---- :  216
An18g03320    : RRLL-YHLYSCQFGTFLFNSEKERAD----WNAKERTRSVWDYFLAR----REQFTNPQYEPQIDDHKRGQERL---- :  612
Fg05432.1     : RRLL-YHLYSCQYGTFLFNSEKQRMD----ARATERTSSVWDYFLCR----RAEFTNPDFDASIDDHVKGQERI---- :  624
Sc_YJR110W    : RRLV-YHLYSCQYGTFLSNSEKEKFQ----QNLPNKTKSVWDYFRSR----RKQFINPNFIQRKRSGMNEHDQNLEEEEKVEW---- :  622
SpNP593789    : RRLL-YHLYSCQYGSFLYNSERERAQ----ASVSTHTRCIWDYFLS----RKDEFKNPNYVPYDDV---- :  526
Lb_143319     : HQLY-YHLYSCQFGTFLYNCERERRVGDG----GPPPCERTISVWDYFNSP----TEMAENKNPAYDPSLDDPMARGPGADMGVLF---- :  557
Um05646.1     : VELH-QVIHECKYGTFLMDCERERFKPQDHDG----RALPALSTRTISAWTYLLRP----DVKNKHRNPNYKPELDDRDPKRKNTDMGVLI---- :  671
ChXP666257    : IIIA-DHLNNSLFCNFHCDNEYERFCID----SRNSTVSLWDTIMYSIKNNSEGNLDNCQVESLLSLNSSIIFHKELVNPLYNPTHTQVRTHADV---- :  666
Pt16054001    : FDLA-YHHLTSLFGTFLCDSFLEIQK----QKIMEQTVSIWSWIMKQ----KDKYINAFYKNPQSIDLDV---- :  509
Pt24407001    : LDLA-YHHLTSLFGTFLCDSFLEIQK----QRIMEQTVSIWSWIMKQ----KDKYINAYYKNPQSIDQDV---- :  512
Pt26750001    : LDLA-YHHMSSLFGSFLCDSFLEIQK----QKVMESTVSIWSWVMKY----KEKYRNAYYLNPGSIEQEV---- :  518
T3692.m00019  : VDLA-FSSYNCQYGTFLANSYQELVK----QDIIKQTISVWSFLLSK----RHIYENPFY--KKDDQIQT---- :  486
T3707.m00240  : SEIA-HHYLSCRFGTFLYNS-YQVHLLEQVA----YNARSQTISLWSYLNSQ----RVTFENPFYLSVHDTNLSI---- :  529
Tp261179C     : LLLS-EHIYSCRFGTLLCDSERERE----VVAGIRQRTYCLWEYLDS----IPELLNPLFDKAYSEAGVL---- :  626
At3g10550C    : VDFM-DCLLSCRFCNFLCNSEKEREQ----CGIADACGCLWAYLTDLRSFSAT----SHVHCNPFYDPLKYDGP---- :  586
At5g04540     : VDFI-DCLLSCRFCNFLCNSEKERQQ----CGISETCGCIWAYLADLRSSSGT----SHVHCNPFYDPSRYDGP---- :  586
O8g44260.1    : VDFM-DCVLSCRFCNFLCNSERERQ----SGAVSSCHCMWTYLADLRASGGS----FHKHRNPFYDPLKHNGP---- :  591
O8g44260.2    : GTLS-NSPNRGALGSSVSTSNTTSGQSQTSNNSSPILLQVIYSFLGALC---- :  513
Pop228298C    : VDLL-DCVLSCRFCNFFCNSEKERQL----VGVSESCGCLWAYLADLRSSEGR----SHVHYNLFYSPLKHKGP---- :  555
Pop420099C    : VDLL-DCVLSCRFCNFFCNSEKERQQ----VGISEACGCLWAYLADLRSSEGR----SHVHCNPFYSPLKHNGP---- :  536
Sorghum       : VDFM-DCVLSCRFCNFLCNSERERQ----SGVTNSCRCMWSYLANLRASGGS----FHEHYNPFYDPVKHNGA---- :  588
035009001C    : VDFL-DCVLSCRFCNFLCNSEKERLQ----CGVSDACGCMWKYLADLRASEGK----YHVHYNLFFDPNRHGSA---- :  591
Phy12645      : VELL-DCVLSCRFCNFLCNSEKERVQ----GGITESTACVWAYLKQLRDQKGR----EHEHYNLFYNSAQNAGV---- :  568
Phy71628      : VDLM-DCVLSCRFCNFLCNSEKDRVL----GGIAESTSCVWAYMKQLRDRRGK----EHEHYNLFYNSSQNAGV---- :  575
```

```
                   *      1940         *      1960         *      1980         *      2000         *      2020         *      2040         *      2060         *      2080         *      2100         *      2120         *      2140
SS            : --------------------------------------------CCCCCCCCCCC----------------------------------------------------------------------------------------------------------------------------------------------------------CCHHHHCCCCC :  513
GL_8210       : -------------------------------------------YLKLNCN-RQLP----------------------------------------------------------------------------------------------------------------------------------------------------------AWPGYWEQHIP :  655
GL_13875      : -------------------------------------------YMHISCSMRKMT----------------------------------------------------------------------------------------------------------------------------------------------------------PWSAYWSQVAA :  770
LmF20.1480    : -------------------------------GGAGGATALADFVLLPNCSSKRLV----------------------------------------------------------------------------------------------------------------------------------------------------------FWEALYTREDA :  791
Tb.1.3300     : -------------------------------------------RISPNCSSKRIV----------------------------------------------------------------------------------------------------------------------------------------------------------FWESFYLRHDG :  649
GL_112811     : --------------------------------------------------SLELS----------------------------------------------------------------------------------------------------------------------------------------------------------SWPTISKFWSL :  603
LmF12.0320    : ERQAFAVYPRSGNGEGASGSLAAGKQPRQRAAAAQSSPSTHTPALLPLGQEGKVVEEEAAMTGGRSYAPQPQAPLPACPAAPLYLYDFDCTTQWGVPVASAAPPLRSGQALPSPFSISSSATSPRAHSARPQGEASLAASGRQTQHPHQQAGCNASGATAADEFSRRLLPISYDALYTSGYLNPNFSLRHNRVNVGPLVDFILPSQLTLWRAAYTRHSF : 1335
Tb.6.870      : ------------------------------------------ATTSPCSHPIPLQS-------------------------------------------------------------------------------------------------------------------------------------------FFKVGYGGSSIYTPNWCPAVGKFSS :  681
Dd0186027     : ------------------------------------------AFHLPEWRSEHIT----------------------------------------------------------------------------------------------------------------------------------------------------------LWKGYYLRYWR :  545
Dd0190143     : --------------------------------------------IKPKLYSRCLE----------------------------------------------------------------------------------------------------------------------------------------------------------LWKACYMRVES :  558
Dd0190271     : --------------------------------------------IRPKLYSRCLE----------------------------------------------------------------------------------------------------------------------------------------------------------LWKSCYMRFES :  553
Dd0204242     : ---------------------------------------LKSSTIFPIADIKLIK----------------------------------------------------------------------------------------------------------------------------------------------------------FWESYYCRSNS :  608
Dd0216866     : -----------------------------------------------------------------------------------------------------------------------------------------------------------------------------------------------------------------FWSNYYMRWNE :  556
Dd0231579     : ---------------------------------------------PIRIDTSELS----------------------------------------------------------------------------------------------------------------------------------------------------------LWSEYYLHWRN :  547
Dd0238869     : --------------------------------------------LIPSIAIPYFS----------------------------------------------------------------------------------------------------------------------------------------------------------LWKDYYLKYLL :  707
Dd0238873     : -------------------------------------------VIFPDCSIKRFK----------------------------------------------------------------------------------------------------------------------------------------------------------VWKQLYFNQSD :  539
Dd0266510     : ---------------------------------------LTATTSIPLTNSTTLDQQLQFKNNNDDGVLFPNPKG------------------------------------------------------------------------------------------------------------------------------------VQLWSDYFLKWRN :  674
EHI_016430    : ------------------------------------------LNLSFLEKQVPV-----------------------------------------------------------------------------------------------------------------------------------------------------------LWRKYYLQYHK :  569
EHI_024380    : ------------------------------------------IDLN-FNVHEYV-----------------------------------------------------------------------------------------------------------------------------------------------------------VWNKYYLRETK :  569
EHI_031310    : ------------------------------------------LPLDVIKQKQPI-----------------------------------------------------------------------------------------------------------------------------------------------------------FWYNYYGSYST :  551
EHI_049780    : ------------------------------------------LTLN-TSLLCIS-----------------------------------------------------------------------------------------------------------------------------------------------------------SWLNYFIRYSQ :  529
EHI_070120    : ------------------------------------------NKLHLNGIPKFV-----------------------------------------------------------------------------------------------------------------------------------------------------------LWEKYYFCNRK :  581
EHI_087780    : ------------------------------------------IETK-FEVFEYR-----------------------------------------------------------------------------------------------------------------------------------------------------------IWNEYYLREFD :  573
EHI_104710    : ------------------------------------------IKVSLNPLQFK------------------------------------------------------------------------------------------------------------------------------------------------------------LWKEYYLRYDK :  583
EHI_161190    : ------------------------------------------LILE-DKQPKLI-----------------------------------------------------------------------------------------------------------------------------------------------------------LWTKYFTNTKL :  560
Dd0191503     : --------------------------------------------SSSSSSSSSLSNGYNSSVQHLKCLRVFQDT---------------------------------------------------------------------------------------------------------------------------------------MWNEYFFRYCF :  549
Dd0191512     : ------------------------------------------M-IKPSCDNDQII----------------------------------------------------------------------------------------------------------------------------------------------------------LWSSFFTEFIN :  942
EHI_048230    : ------------------------------------------IEIPCTKLHLNNLFST-------------------------------------------------------------------------------------------------------------------------------------------------------IINPYTYNFFT :  495
EHI_078170    : ------------------------------------------YNENLFSVQSLVLLFL-------------------------------------------------------------------------------------------------------------------------------------------------------KWMNVGKNCNF :  519
EHI_107230    : ------------------------------------------IPLQCIKVHFCNEIFC-------------------------------------------------------------------------------------------------------------------------------------------------------QVQTYNPQTVA :  479
EHI_135010    : ------------------------------------------YCNKELPLVHCPYFFF-------------------------------------------------------------------------------------------------------------------------------------------------------RWMNYTYKQTD :  534
EHI_137960    : ------------------------------------------LKNELIEGDYCALIY--------------------------------------------------------------------------------------------------------------------------------------------------------KWLNYSKKCTK :  424
EHI_141820    : ------------------------------------------WPQSIVLLQPNLFI---------------------------------------------------------------------------------------------------------------------------------------------------------DFINYTKDKNP :  480
EHI_151670    : ------------------------------------------LIFKPYKCYYSFD----------------------------------------------------------------------------------------------------------------------------------------------------------KWLGYSKDNIS :  478
EHI_185230    : ------------------------------------------MYLVIPECPCCILFD------------------------------------------------------------------------------------------------------------------------------------------------------FDEWMNFNKEAVC :  486
EHI_197200    : ---------------------------------------------------------------------------------------------------------------------------------------------------------------------------------------------------------------------------- :    -
EHI_140980    : ------------------------------------------VSIS-ENLLTIP-----------------------------------------------------------------------------------------------------------------------------------------------------------LWTKYYLRYSF :  500
EHI_188050    : ------------------------------------------TFFPIFDMSEFS-----------------------------------------------------------------------------------------------------------------------------------------------------------VWNEYYFRNAS :  587
MTM1_Hu       : --------------------------------------------LYPVASMRHLE----------------------------------------------------------------------------------------------------------------------------------------------------------LWVNYYIRWNP :  512
MTMR1_Hu      : --------------------------------------------LYPVASLSHLE----------------------------------------------------------------------------------------------------------------------------------------------------------LWVNYYVRWNP :  512
MTMR2_Hu      : --------------------------------------------LYPVASMRHLE----------------------------------------------------------------------------------------------------------------------------------------------------------LWVGYYIRWNP :  513
DrMTMR2       : --------------------------------------------LFPTVGIRHLQ----------------------------------------------------------------------------------------------------------------------------------------------------------LWVSYYIRWNP :  512
N001636543    : --------------------------------------------LFPVPSLRRLE----------------------------------------------------------------------------------------------------------------------------------------------------------LWTRYYIRWNP :  513
Tad38469      : ----------------------------------------------PNASLRYIS----------------------------------------------------------------------------------------------------------------------------------------------------------LWSSYYLRWNP :  507
MTMR3_Hu      : --------------------------------------------LYPVCHVRNLM----------------------------------------------------------------------------------------------------------------------------------------------------------LWSAVYLPCPS :  551
MTMR4_Hu      : --------------------------------------------LHPVCHVRALH----------------------------------------------------------------------------------------------------------------------------------------------------------LWTAVYLPASS :  545
N001631983    : ---------------------------------------------------------------------------------------------------------------------------------------------------------------------------------------------------------------------------- :    -
Tad64213      : ----------------------------------------------PSYEFRDLS----------------------------------------------------------------------------------------------------------------------------------------------------------FWTRMYFPTSS :  565
MTMR5_Hu      : --------------------------------------------LRPYSNVSNLK----------------------------------------------------------------------------------------------------------------------------------------------------------VWDFYTEETLA :  718
DmMTMR5/13    : --------------------------------------------LRPQNNVAALD----------------------------------------------------------------------------------------------------------------------------------------------------------LWCYYTNEELA :  749
DrMTMR5       : --------------------------------------------LKPYSFVSNLK----------------------------------------------------------------------------------------------------------------------------------------------------------VWDFYTEETLS :  712
MTMR13_Hu     : --------------------------------------------LKPNVNVSSLK----------------------------------------------------------------------------------------------------------------------------------------------------------KWDYYIEETLS :  717
Nv109357      : ----------------------------------------------PFCRISNLV----------------------------------------------------------------------------------------------------------------------------------------------------------VWKYYLTENLS :  722
Tad51481      : ----------------------------------------------PVASLTNLK----------------------------------------------------------------------------------------------------------------------------------------------------------VWDYYIKRNLY :  781
M001750622    : --------------------------------------------WRQVYMPTPVLATNEQAGKGSCPDH--------------------------------------------------------------------------------------------------------------------------------------------VWQAAHEHAKS :  642
MTMR6_Hu      : --------------------------------------------LEPNTVSFNFK----------------------------------------------------------------------------------------------------------------------------------------------------------FWRNMYHQFDR :  504
MTMR7_Hu      : --------------------------------------------LHLPTTPCNFMYK--------------------------------------------------------------------------------------------------------------------------------------------------------FWSGMYNRFEK :  509
DrMTMR7       : --------------------------------------------LRPLTTAYCFK----------------------------------------------------------------------------------------------------------------------------------------------------------FWKGMYGRAER :  506
MTMR8_Hu      : --------------------------------------------LNPSTVPYNIQ----------------------------------------------------------------------------------------------------------------------------------------------------------FWCGMYNRFDK :  505
N001626810    : --------------------------------------------LWPDTSPQAIR----------------------------------------------------------------------------------------------------------------------------------------------------------FWRAMYNRYDN :  507
Tad56124      : -------------------------------------------IIIPLINPQCIR----------------------------------------------------------------------------------------------------------------------------------------------------------FWSGMYNRHAF :  506
M01746603C    : --------------------------------------------LNPSLAPQHFE----------------------------------------------------------------------------------------------------------------------------------------------------------LWSAMYCRWES :  482
MTMR9_Hu      : ---------------------------------------------WPSVAPQSLP----------------------------------------------------------------------------------------------------------------------------------------------------------LWEGIFLRWNR :  503
DrMTMR9       : ---------------------------------------------WPSVAPQSLL----------------------------------------------------------------------------------------------------------------------------------------------------------LWEGVFLRWNR :  503
N001625874    : ---------------------------------------------WPSVAPQSLV----------------------------------------------------------------------------------------------------------------------------------------------------------LWPGLYLKWQK :  505
Tad28926      : --------------------------------------------TVPHCIVCMKIY---------------------------------------------------------------------------------------------------------------------------------------------------------LCHYNVYEWSS :  507
M01749550C    : --------------------------------------------ICPSPRPQAIV----------------------------------------------------------------------------------------------------------------------------------------------------------LWRNLYC-WR- :  334
MTMR10_Hu     : --------------------------------------------HNPFYIGKSTPCIQNGSVK--------------------------------------------------------------------------------------------------------------------------------------------------SFKRTKKSYSS :  500
DrMTMR10      : --------------------------------------------LLPQLLPSHLS----------------------------------------------------------------------------------------------------------------------------------------------------------VWKLYFLRWVP :  611
MTMR11_Hu     : --------------------------------------------QNPGYDPEHCPD---------------------------------------------------------------------------------------------------------------------------------------------------------SWLPRPQPSFM :  481
MTMR12_Hu     : --------------------------------------------LLPHIEGPEIK----------------------------------------------------------------------------------------------------------------------------------------------------------VWAQRYLRWIP :  609
N001629628    : --------------------------------------------KNPMNGDNEIEEIYSRVNPTAPAMK--------------------------------------------------------------------------------------------------------------------------------------------FWSGCYLRWLP :  530
Tad54716      : -----------------------------------------KTIAKPLTVVTDIFDMK-------------------------------------------------------------------------------------------------------------------------------------------------------IWHSLYLRSFG :  592
M01745983C    : --------------------------------------------IMPTCSLKRIE----------------------------------------------------------------------------------------------------------------------------------------------------------LWSDYFLRYDA :  671
Tad51955      : ------------------------------------------IVWVPDVRATECYDCKQRFTAI---------------------------------------------------------------------------------------------------------------------------------------------RRKVMWTSFLQDVHP :  725
Mbrevi5R3     : ---------------------------------------------------------------------------------------------------------------------------------------------------------------------------------------------------------------------------- :    -
Tad62282      : --------------------------------------------LAELFQIKPIRKVMLKKNERNINLEYEEEDNPQSWKTAPNIDLHYSNESLTDSNGTPDNSLRRQTI---------------------------------------------------------------------------------------------------NFVYILQRKGS :  503
Nv82921       : ----------------------------------------------PVSSLRRLQ----------------------------------------------------------------------------------------------------------------------------------------------------------LWSDYFLRYD- :  235
An18g03320    : --------------------------------------------IFPRINE--VR----------------------------------------------------------------------------------------------------------------------------------------------------------WWSESFGRTDA :  632
Fg05432.1     : --------------------------------------------LLPRLKE--IR----------------------------------------------------------------------------------------------------------------------------------------------------------WWHQVFGRTED :  644
Sc_YJR110W    : --------------------------------------------ISPDLKK--VQ----------------------------------------------------------------------------------------------------------------------------------------------------------WWWQLYGRKDS :  642
SpNP593789    : --------------------------------------------IMPDPSS--LR----------------------------------------------------------------------------------------------------------------------------------------------------------WWSASFAQPDE :  546
Lb_143319     : --------------------------------------------VNPKD----VR----------------------------------------------------------------------------------------------------------------------------------------------------------FWHELYGRTDE :  575
Um05646.1     : --------------------------------------------ADSRD----VR----------------------------------------------------------------------------------------------------------------------------------------------------------YFEQLFQRASG :  689
ChXP666257    : --------------------------------------------LILDSSSFGIT----------------------------------------------------------------------------------------------------------------------------------------------------------PWMPFWFRFTP :  688
Pt16054001    : --------------------------------------------IIPDSFTGKSVT---------------------------------------------------------------------------------------------------------------------------------------------------------FWKEYFLYYSL :  532
Pt24407001    : --------------------------------------------VIPEQFTGKSIT---------------------------------------------------------------------------------------------------------------------------------------------------------FWKEYFLYYSL :  535
Pt26750001    : --------------------------------------------IIPEQFTGKSVK---------------------------------------------------------------------------------------------------------------------------------------------------------FWQEYFLNYSS :  541
T3692.m00019  : --------------------------------------------LKPD-FQQRSLK---------------------------------------------------------------------------------------------------------------------------------------------------------IWNRYFFFYQF :  508
T3707.m00240  : --------------------------------------------IFPDSHT-RRFS---------------------------------------------------------------------------------------------------------------------------------------------------------VWRDHFMIYQP :  551
Tp261179C     : -------------------------------------------LMPLPMLLRNVT----------------------------------------------------------------------------------------------------------------------------------------------------------LWTDRLCMYGS :  649
At3g10550C    : --------------------------------------------LLPPAASLAPT----------------------------------------------------------------------------------------------------------------------------------------------------------LWPQFHLRWAC :  608
At5g04540     : --------------------------------------------LLPPAAALAPT----------------------------------------------------------------------------------------------------------------------------------------------------------LWPQFHLRWAC :  608
O8g44260.1    : --------------------------------------------LVPPAAALAPT----------------------------------------------------------------------------------------------------------------------------------------------------------LWPQFYLRWTC :  613
O8g44260.2    : ---------------------------------------------------------------------------------------------------------------------------------------------------------------------------------------------------------------------------- :    -
Pop228298C    : --------------------------------------------LLPPAAALAPT----------------------------------------------------------------------------------------------------------------------------------------------------------LWPQFHLRWAC :  577
Pop420099C    : --------------------------------------------LLPPAAALAPT----------------------------------------------------------------------------------------------------------------------------------------------------------LWPQFHLRWAC :  558
Sorghum       : --------------------------------------------LLPPAAALAPT----------------------------------------------------------------------------------------------------------------------------------------------------------LWPQFYLRWTC :  610
O35009001C    : --------------------------------------------ILPPAAALAPT----------------------------------------------------------------------------------------------------------------------------------------------------------LW--------- :  604
Phy12645      : --------------------------------------------LLPPAAATAPS----------------------------------------------------------------------------------------------------------------------------------------------------------LWRQYFLRWSC :  590
Phy71628      : --------------------------------------------LLPPAAASAPS----------------------------------------------------------------------------------------------------------------------------------------------------------LWRQYFLRWSC :  597
```
