# Supplementary material for: A phylogenetic survey of myotubularin genes of eukaryotes: distribution, protein structure, evolution, and gene expression
Source: BMC Evol Biol. 2010 Jun 24;10:196. doi: 10.1186/1471-2148-10-196 (PMC2927912; doi:10.1186/1471-2148-10-196)
Supplement: Additional file 2 — Predicted Nuclear Localization Signals (NLS) in Animal Myotubularin Homologue Sequences. This file presents data summarizing predicted nuclear localization signals (NLS) in metazoan myotubularin homologue sequences of the R5 clade. [file 1471-2148-10-196-S2.PDF]

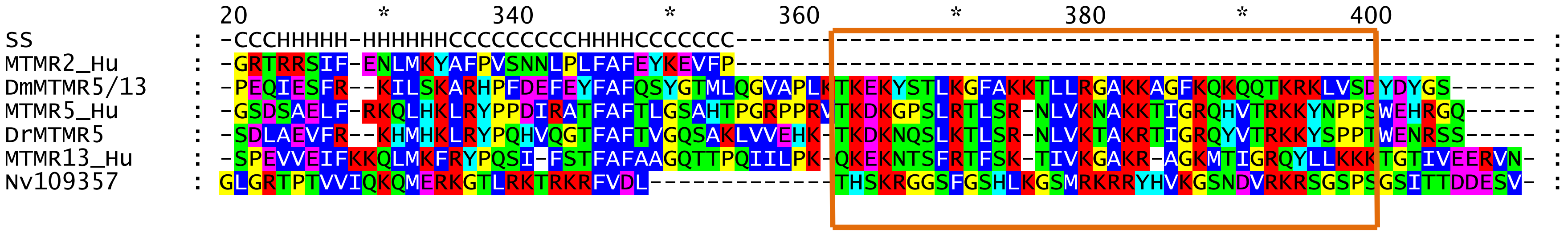

Legend Additional File 2: Predicted Nuclear Localization Signals (NLS) in Animal Myotubularin Homologue Sequences

Myotubularin sequences were analyzed for nuclear localization signals (NLS) as detailed in Methods. Positive predictions were obtained for: DmMTMR5/13 (1089 - KKTLLRGAKKAGFKQKQQ – 1106) [2 State HMM Static, 2 State HMM Dynamic]; MTMR5_Hu (1058 - NAKK – 1061) [2 State HMM Dynamic]; MTMR13_Hu (1048 - KRAGKMTIGR – 1057) [4 State HMM Static]; Nv109357 (1050 - KGTLRKTRKRFVDLTHSKRGGSFGSHLKGSMRKRRYHVKGSNDVRKR – 1096) [2 State HMM Static, 2 State HMM Dynamic]. The location of a conserved basic region is indicated by the orange box. The sequence and secondary structure (“SS”) of human MTMR2 (PDB: 1LW3) [1] is given for orientation. The conserved basic region lies between α2 and α3 of the solved structure (positions 362 to 400 in the reference alignment [Additional File 1]). In deuteration studies of human MTMR2 [2] this is a solvent-accessible region, consistent with availability for interactions with nuclear import proteins.
Sequence names: “Dr” (Danio rerio); “Dm” (Drosophila melanogaster); “Hu” (Homo sapiens); “Nv” (Nematostella vectensis).

1. Begley MJ, Taylor GS, Kim SA, Veine DM, Dixon JE, Stuckey JA: **Crystal structure of a phosphoinositide phosphatase, MTMR2: insights into myotubular myopathy and Charcot-Marie-Tooth syndrome.** *Mol Cell* 2003, **12:**1391-1402.
2. Begley MJ, Taylor GS, Brock MA, Ghosh P, Woods VL, Dixon JE: **Molecular basis for substrate recognition by MTMR2, a myotubularin family phosphoinositide phosphatase.** *Proc Natl Acad Sci U S A* 2006, **103:**927-932.
