# Supplementary material for: A phylogenetic survey of myotubularin genes of eukaryotes: distribution, protein structure, evolution, and gene expression
Source: BMC Evol Biol. 2010 Jun 24;10:196. doi: 10.1186/1471-2148-10-196 (PMC2927912; doi:10.1186/1471-2148-10-196)
Supplement: Additional file 3 — Predicted Nuclear Localization Signals (NLS) in Plant Myotubularin Homologue Sequences. This file presents data summarizing predicted nuclear localization signals (NLS) in plant myotubularin homologue sequences. [file 1471-2148-10-196-S3.PDF]

```
                  *          300          *          320
SS          : HHHHHHHHHHHHCCCCCCC----EEEEECCCHHHHHHH :
MTMR2_Hu    : KEDEKYLQAIMDSNAQSHK----IFIFDARPSVNAVAN :
At3g10550C  : NLDEKLVAAFCSQLPGAKGERRKLYIADARPRKNALAN :
At5g04540   : NSDEKLVASFCTQLAGHKGARRKLYIVDARPRKNALAN :
O8g44260.1  : NTDEKLVSALCTQIIDATGSLRKLYIVDARPRANALAN :
O8g44260.2  : NTDEKLVSALCTQIIDATGSLRKLYIVDARPRANALAN :
Pop228298C  : NTDEKLVAALCSQLGGDKKGRRKLYIADARPRKNALAN :
Pop420099C  : NTDEKLVAELCSQLGDEKKRRRKLYIADARPRKNALAN :
Sorghum     : NADEKLVCALSSRTTDEKGSPRKLYIIDARPRANALAN :
035009001C  : NTDEKIVAALCTQLAGARETRRKLYIADARPRKNALAN :
Phy12645    : KEDEQLVAAVCNTA----NQRRKLYIADARPRKNALAN :
Phy71628    : TADEKLVAAVCDTI----NQRRKLYIADARPRKNALAN :
```

Legend Additional File 3: Predicted Nuclear Localization Signals (NLS) in Plant Myotubularin Homologue Sequences

Plant myotubularin homologue sequences were analyzed for nuclear localization signals (NLS) as detailed in Methods. Positive predictions were obtained for: Pop420099C (227 - KKRRRK – 232) [2 State HMM Static, 2 State HMM Dynamic] and Pop228298C (214 - KKGRRKLYIADARPRK – 229) [4 State HMM Static]. The location of a conserved basic region is indicated by the orange box. The sequence and secondary structure (“SS”) of human MTMR2 (PDB: 1LW3) [1] is given for orientation. The conserved basic region lies between α5 and α6 of the solved structure (positions 780 to 882 of the reference alignment [Additional File 1]). In deuteration studies of human MTMR2 [2] this is a solvent-accessible region, consistent with availability for interactions with nuclear import proteins. Sequence names: “At” (Arabidopsis thaliana); “O8g” (Oryza sativa); “Pop” (Populus trichocarpa); “Sorghum” (Sorghum bicolor); “0” (Vitis vinifera); “Phy” (Physcomitrella patens).

1. Begley MJ, Taylor GS, Kim SA, Veine DM, Dixon JE, Stuckey JA: **Crystal structure of a phosphoinositide phosphatase, MTMR2: insights into myotubular myopathy and Charcot-Marie-Tooth syndrome.** *Mol Cell* 2003, **12:**1391-1402.
2. Begley MJ, Taylor GS, Brock MA, Ghosh P, Woods VL, Dixon JE: **Molecular basis for substrate recognition by MTMR2, a myotubularin family phosphoinositide phosphatase.** *Proc Natl Acad Sci U S A* 2006, **103:**927-932.
