# Supplementary material for: A phylogenetic survey of myotubularin genes of eukaryotes: distribution, protein structure, evolution, and gene expression
Source: BMC Evol Biol. 2010 Jun 24;10:196. doi: 10.1186/1471-2148-10-196 (PMC2927912; doi:10.1186/1471-2148-10-196)
Supplement: Additional file 6 — Additional Information on ROCO Sequence Alignment. This file presents species designations and database accession numbers for sequences presented in the multiple sequence alignment of Figure 4. [file 1471-2148-10-196-S6.DOCX]

Additional File 6: Additional Information on ROCO Protein Domain Multiple Sequence Alignment (Figure 4)

ROCO domain reference proteins:

“Ct_ROCO” (*Chlorobium tepidum* [PDB:3dpu_A])

“Ns_LRRP1” (*Nostoc* sp. PCC 7120 [GenBank:NP_484168.1])

“Mb_ROCO1” (*Methanosarcina barkeri* str. Fusaro [GenBank:YP_306802.1])

“Hs_LRK1” (*Homo sapiens*) [GenBank:AAY67799.1]

“Hs_LRRK2” (*Homo sapiens*) [Swiss-Prot:Q5S007.1]

“Ce_LRK1” (*Caenorhabditis elegans*) [GenBank:NP_492839.4]

The species prefixes are as follows:

“Tad” (*Trichoplax adhaerens*); “Nve” (*Nematostella vectensis*); “Dd” (*Dictyostelium discoideum*); “Mbr” (*Monosiga brevicollis*); “EHI” (*Entamoeba histolytica*).

Several *Dictyostelium* sequences are referred to by their gene names. The DictyBase (<http://dictybase.org>) reference numbers for these sequences is as follows: “Dd_pats1” (DDB0191503); “Dd_roco4” (DDB0191509); “Dd_roco11” (DDB0191297); “Dd_qkgA-1” (DDB0185215); “Dd_qkgA-2” (DDB0304847); “Dd_roco5” (DDB0232931); “Dd_roco6” (DDB0214834); “Dd_gbpC” (DDB0191359); “Dd_roco8” (DDB0191480); “Dd_roco10” (DDB0201665); “Dd_roco7” (DDB0191295); “Dd_roco9” (DDB0191512).
